# Patient Information and Surgery Decisions: A Discrete Choice Experiment

**DOI:** 10.1002/hec.70135

**Published:** 2026-07-19

**Authors:** Francesca Cornaglia, Alessandro Iaria, Eugenio Felipe Merlano, John R. Prowle

**Affiliations:** ^1^ Queen Mary University of London London UK; ^2^ University of Bristol Bristol UK; ^3^ William Harvey Research Institute Queen Mary University of London London UK

**Keywords:** decision support systems, incorrect beliefs, shared decision‐making, treatment availability, welfare loss

## Abstract

Surgical treatments are offered more widely than ever before, increasingly to older and high‐risk patients. However, it is unclear whether patients making surgical decisions are fully informed about the risks of surgery and the available non‐surgical treatments. Especially for high‐risk patients, unrecognized elevated post‐surgery risks may make non‐surgical treatments particularly relevant. In this paper, we study the role of information provision and the availability of non‐surgical treatments for high‐risk patients considering hip replacement surgery in the UK. We develop a discrete choice model of incorrect beliefs about post‐surgery outcomes and limited availability of non‐surgical treatments, which we estimate using an online choice experiment. In the experiment, participants take the perspective of a hypothetical high‐risk patient; we elicit their beliefs about post‐surgery outcomes and randomize additional information about these outcomes and the availability of physiotherapy. We find that beliefs differ systematically from predicted post‐surgery outcomes and that the provision of additional information has heterogeneous effects on belief accuracy. We show that excluding physiotherapy from the available treatments accounts for four‐fifths of the participants' total welfare loss, while incorrect beliefs about post‐surgery outcomes account for the remainder. We find evidence that education is a strong predictor of welfare loss from incorrect beliefs.

## 1 Introduction

Surgical treatments are offered more widely than ever before, increasingly to older and high‐risk patients (S. Shaw et al. 2020). This is particularly true for elective procedures, in which patients and clinicians engage in shared decision‐making. UK General Medical Council guidelines state that shared decision‐making requires informing patients of all material risks of a procedure and of the reasonable alternative treatments available before they consent to surgery (General Medical Council 2020). In the British National Health Service (NHS), approximately 250,000 high‐risk patients—increasingly older and affected by chronic disease—undergo major surgery each year (OSIRIS 2018); this group accounts for the large majority of deaths after surgery (R. M. Pearse et al. 2006). Even when surgery and anesthesia are straightforward, roughly one in three of these patients develops serious medical complications that delay recovery, prolong hospital stays, and reduce long‐term quality of life and survival (OSIRIS 2018; ISOS 2016; Fowler et al. 2023). Surgery typically remains the default treatment, and regret about the decision is not unusual (S. E. Shaw et al. 2023; Doherty and Saunders 2013). Despite these stakes, most doctors do not feel prepared to meet shared decision‐making standards for complex high‐risk patients, for whom the risks and benefits of surgery are most uncertain (OSIRIS 2018; Bougeard et al. 2017).

In this paper, we study how two aspects of the shared decision‐making environment shape the choices of high‐risk patients considering hip replacement in the UK: information about likely post‐surgery outcomes and the availability of physiotherapy as a non‐surgical treatment. These aspects are closely related for high‐risk patients: when surgical risks are elevated, more accurate information about those risks may change the expected value of surgery relative to non‐surgical management, and physiotherapy may become a particularly relevant non‐surgical alternative. In addition, because surgery is typically the default treatment, the choice of surgery alone cannot distinguish a strong preference for it from the limited availability of non‐surgical treatments at the point of the surgical decision. To investigate these aspects, we develop a discrete choice model of incorrect beliefs about post‐surgery outcomes and limited availability of non‐surgical treatments, which we estimate using an online choice experiment. In the experiment, we elicit beliefs about post‐surgery outcomes and randomize both the provision of additional information about these outcomes and the availability of physiotherapy. The resulting welfare calculations measure the patient losses associated with incorrect beliefs and with the exclusion of physiotherapy from the available treatments, providing evidence that can inform shared decision‐making when guidance on what is material to individual patients remains limited.

From an economic theory perspective, the decision to undergo surgery depends on whether the expected benefit exceeds the expected cost, yet uncertainty about experienced post‐surgery outcomes makes decisions significantly more complex for older and high‐risk patients (Arrow 1963). The information problem we study is on the patient side: patients lack complete information about their likely post‐surgery outcomes, and this incomplete information is compounded by the fact that surgical decisions are typically made once, are irreversible, and patients cannot learn by doing through repeated exposure (Crawford and Shum 2005; Erdem and Keane 1996). Moreover, few doctors have the breadth of expertise needed to predict every likely long‐term outcome for complex surgical patients with multiple comorbid diseases (Hunink et al. 2014). Consequently, high‐risk patients frequently consent to surgery without recognising that poor long‐term outcomes may outweigh the potential benefits. Patient opinions about high‐burden treatments vary widely, and quality of life is valued above quantity of life in some situations (Clarke et al. 2008; Coulman et al. 2016; Husain et al. 2008; Lytvyn et al. 2016). When the risk of poor post‐surgery outcomes is elevated, the information patients receive about those outcomes and the availability of alternative non‐surgical treatments become central to patient welfare.

To evaluate the welfare consequences of these two channels, we design an online experiment and estimate a discrete choice model building on Train (2015). In the experiment, participants take the position of a high‐risk patient facing a hip‐replacement decision, and we randomise two independent interventions in a 2×2 design. The first intervention, the *information intervention*, concerns the information provided to participants about three post‐surgery outcomes: the probabilities of extended hospital stay, hospital readmission within 6 months, and death within 90 days. All participants receive the qualitative risk information of a standard NHS consultation, while a randomly selected group also receives additional quantitative information from the NHS Hospital Episode Statistics (HES) about the three post‐surgery outcomes. The second intervention, the *treatment availability intervention*, randomly assigns participants to one of two sets of treatments: a constrained set of surgery and GP‐led management, or a full set that additionally includes physiotherapy as a non‐surgical alternative. After random assignment of the two interventions, but before treatment choices are made, we elicit participants' beliefs about the three post‐surgery outcomes. Because we elicit beliefs directly and randomise both interventions, the experiment allows the identification of the model while remaining agnostic about belief formation, and allows us to evaluate the welfare effects of incorrect beliefs and limited treatment availability separately from unobserved preferences.

This paper is part of a multidisciplinary research programme on shared decision‐making for patients considering major surgery, funded by the National Institute for Health and Care Research (NIHR) (R. Pearse et al. 2024). The experimental scenarios were co‐designed with clinical researchers experienced in perioperative medicine and critical care, drawing on epidemiological descriptions of the UK surgical population based on HES (Fowler et al. 2022, 2023) and publicly available data on patient‐reported outcomes after hip surgery (Vickery et al. 2025). The experimental design also relates to the broader application of statistical learning and AI in clinical practice (Secinaro et al. 2021; Yu et al. 2018). The information intervention mimics the use of predictive models in the form of clinical decision support systems to assist doctors and patients in shared decision‐making (Amann et al. 2020), in a setting where such systems complement, rather than substitute for, medical practitioners (Sezgin 2023).

Our online experiment and model estimates yield three main findings. First, participants' elicited beliefs about post‐surgery outcomes differ systematically from the predicted probabilities computed from HES data for the high‐risk clinical profile used in the experiment, and the information intervention has heterogeneous effects on belief accuracy: when participants underestimate the predicted probabilities, as for extended hospital stay, it moves their beliefs closer to the predictions, but when they already overestimate the predicted probabilities, as for hospital readmission within 6 months and death within 90 days, it amplifies overestimation. Second, the information intervention reduces the share of participants choosing surgery, especially when physiotherapy is not available, while the inclusion of physiotherapy substantially changes the composition of non‐surgical choices away from GP‐led management. Third, in our experimental setting, our welfare calculations show that excluding physiotherapy from the available treatments accounts for the large majority (around four‐fifths) of average total welfare loss, while incorrect beliefs about post‐surgery outcomes account for the remainder. The estimated welfare losses are highly heterogeneous across participants. Underlying this heterogeneity, we find that education is a strong predictor of the welfare loss from incorrect beliefs, with more‐educated participants holding beliefs closer to the predictions and incurring smaller losses.

This paper makes three contributions. First, it contributes to the growing literature on shared decision‐making and decision support systems. Papers in this literature have examined the theoretical interaction of predictions and decision‐making (Agrawal et al. 2019; Athey et al. 2020) and the empirical implications of skilled experts in facilitating consumer choice of complex products such as health insurance (Gruber et al. 2020). We study how elicited beliefs are influenced by the provision of additional quantitative information about post‐surgery outcomes within a clinical consultation setting. This setting is distinctive in that information complements expert recommendations, post‐surgery outcomes are uncertain for both patients and doctors, and decisions are irreversible and rarely repeated.

Second, the paper contributes to the literature on choice constraints and the availability of alternatives in health economics. Earlier research has explored the effects of expanding choice of hospitals and health insurance plans (Dafny et al. 2013; Gaynor et al. 2016) and the role of consideration sets in medical decisions (Fiebig et al. 2017). We study the effects on patient welfare of experimentally expanding the set of potential treatments by adding a non‐surgical alternative in the case of a widely performed surgical procedure. This margin is particularly relevant in our setting, as non‐surgical alternatives play a vital policy role for high‐risk patients whose elevated chances of post‐surgery complications carry substantial medical risks and quality‐of‐life consequences (Ludwiczak et al. 2024).

Finally, this paper contributes to the literature on welfare calculations in discrete choice models when elicited and experienced attributes differ. In our choice experiment, we elicit participants' beliefs directly rather than inferring them or assuming their distribution. We also randomise the information participants receive, which makes it exogenous and breaks the potential correlation between belief formation and unobserved preferences. Previous papers that elicited beliefs in choice models (Arcidiacono et al. 2012; Attanasio and Kaufmann 2009) typically did not leverage exogenous variation in the information used by decision makers to form beliefs. We extend the estimation of welfare effects to a context in which both elicited and experienced attributes differ (Allcott 2013; Schmeiser 2014; Train 2015) and the choice set varies, allowing the interaction between these two sources of welfare to be studied in a unified setting.

The rest of the paper is organised as follows. Section 2 outlines the discrete choice model of incorrect beliefs and limited treatment availability that we estimate using the online experiment introduced in Section 3. Section 4 describes the experimental data collected, and Section 5 discusses the main findings. Section 6 concludes.

## 2 A Model of Incorrect Beliefs and Limited Availability of Treatments

We study the role of information provision and the availability of non‐surgical treatments in the context of hip replacement surgery. We use the term “treatment” to refer to the healthcare options available to patients and the term “intervention” for our experimental manipulations, that is, the provision of profile‐specific forecasts of post‐surgery outcomes and changes to the set of non‐surgical treatments. We distinguish between “benchmark” or “predicted” values of post‐surgery outcomes, which are calculated from NHS Hospital Episode Statistics (HES) for patients matching the clinical profile used in our experiment, and “expected” or “elicited” values, which reflect patients' stated beliefs about these post‐surgery outcomes.

We now introduce our discrete choice framework and notation, while we describe in detail all interventions, treatments, and variables in Section 3. Patients choose among up to three treatments: hip replacement surgery (S) and two non‐surgical alternative treatments, GP‐led management (G) and physiotherapy (P). We define J={S,G,P} as the full set of treatments from which a patient can choose. In our experiment, we also consider an intervention in which some patients face a constrained set of treatments C={S,G} that does not include physiotherapy, one of the non‐surgical treatments.

We denote *benchmark* post‐surgery outcomes as the vector $c_{S}$. These are relevant only for surgery. The benchmark post‐surgery outcomes include the probabilities of extended hospital stay, hospital readmission within 6 months, and death within 90 days. Building on Train (2015), we consider a discrete choice model in which patients choose among treatments and have potentially incorrect beliefs about the benchmark post‐surgery outcomes $c_{S}$. They make choices on the basis of *expected* post‐surgery outcomes ${\tilde{c}}_{iS}$ that may turn out to be different from the benchmark post‐surgery outcomes $c_{S}$.

The *benchmark* indirect utility patient i obtains from choosing each treatment is:

(1) $$\begin{array}{c} U_{iS}=V_{iS}\left(c_{S}\right)+\varepsilon_{iS}\\ =\alpha \;w_{iS}+\beta^{\prime }c_{S}+\varepsilon_{iS},\\ U_{ik}=V_{ik}+\varepsilon_{ik}\;\;\;\;\text{for}\;k\;\in \;\left\{G,P\right\}\\ =\delta_{k}+\alpha \;w_{ik}+\varepsilon_{ik}, \end{array}$$

where $V_{ij}$ denotes the systematic component of indirect utility and $\varepsilon_{ij}$ the residual portion of indirect utility; $\delta_{j}$ is a treatment‐specific constant (relative to $\delta_{S}$, which is normalized zero), $w_{ij}$ is the waiting time to obtain treatment j, $c_{S}$ is the vector of benchmark post‐surgery outcomes, (α,β) are preference parameters with α<0.

The set of treatments A∈{J,C}, waiting time $w_{ij}$, and benchmark post‐surgery outcomes $c_{S}$ are assumed to be independent of $\varepsilon_{ij}$ and thus exogenous.^1^ The patient knows $\left(\delta_{j},\alpha ,\beta ,w_{ij},\varepsilon_{ij},A\right)$ but may not know the benchmark post‐surgery outcomes $c_{S}$ and makes decisions based on her *expectation* of $c_{S}$. Patient i forms this expectation given her available information $I_{i}$ about the benchmark post‐surgery outcomes:

(2) $${\tilde{c}}_{iS}\left(I_{i}\right)=\int c_{S}\;dB_{i}\left(c_{S}|I_{i}\right),$$

where $B_{i}\left(\cdot |I_{i}\right)$ is the distribution of i’s beliefs about $c_{S}$. Given (2), patient i’s *expected* indirect utility from hip replacement surgery is:

(3) $$\begin{array}{c} EU_{iS}=V_{iS}\left({\tilde{c}}_{iS}\left(I_{i}\right)\right)+\varepsilon_{iS}\\ =\alpha \;w_{iS}+\beta^{\prime }{\tilde{c}}_{iS}\left(I_{i}\right)+\varepsilon_{iS}, \end{array}$$

where everything is the same as in the first two rows of (1) with the only exception of the expected post‐surgery outcomes ${\tilde{c}}_{iS}\left(I_{i}\right)$, which may or may not be equal to the benchmark post‐surgery outcomes $c_{S}$. For k∈{G,P}, we instead have $EU_{ik}=U_{ik}$, where $U_{ik}$ is as in the second two rows of (1).

The econometrician does not observe $\varepsilon_{i}={\left(\varepsilon_{ij}\right)}_{j\in J}$ but is assumed to know its distribution across patients. We assume that the vector $\varepsilon_{i}$ is independent of $I_{i}$ and follows a Generalized Extreme Value (GEV) distribution that gives rise to a nested logit model. Let $H=\left\{H_{S},H_{C}\right\}$ be the partition of J into the (singleton) invasive nest $H_{S}=\left\{S\right\}$ and the conservative (non‐surgical) nest $H_{C}=\left\{G,P\right\}$.^2^ This allows $\varepsilon_{iG}$ and $\varepsilon_{iP}$ to be correlated, and so the conservative treatments G and P to share similar unobserved features. The nesting parameter λ∈(0,1] governs the degree of correlation between $\varepsilon_{iG}$ and $\varepsilon_{iP}$: when λ=1, the model reduces to the multinomial logit, which does not allow for any correlation among the $\varepsilon_{ij}$’s.

Although the assumption that $\varepsilon_{i}$ follows a GEV distribution is standard, the assumption that $\varepsilon_{i}$ is independent of $I_{i}$ is not. In observational data, patients with different unobserved determinants of treatment choice may gather different information. Together with the fact that the expectations ${\tilde{c}}_{iS}\left(I_{i}\right)$ are generally unobserved, this creates two limitations for estimation: the econometrician may not observe the beliefs that enter patient choices, and the information set on which those beliefs are based may be endogenous to unobserved determinants of choice. As we discuss in Section 2.1, addressing these two limitations is a special feature of our experiment.

Given these assumptions, the nested‐logit treatment choice probabilities take the following form. For A∈{J,C} and $x\in \left\{{\tilde{c}}_{iS},c_{S}\right\}$, define:

$$D_{i}\left(x,A\right)=\exp\left(V_{iS}\left(x\right)\right)+{\left(\sum_{k\in A\cap H_{C}}\exp\left(\frac{V_{ik}}{\lambda }\right)\right)}^{\lambda }.$$

Then, given $x\in \left\{{\tilde{c}}_{iS},c_{S}\right\}$, patient i’s choice probability for treatment j∈A is:

(4) $$\mathrm{Pr}\left[j|A,x\right]=\left\{\begin{array}{cc} \frac{\exp\left(V_{iS}\left(x\right)\right)}{D_{i}\left(x,A\right)}, & \text{if}\;j=S,\\ \frac{\exp\left(\frac{V_{ij}}{\lambda }\right){\left(\sum_{k\in A\cap H_{C}}\;\exp\left(\frac{V_{ik}}{\lambda }\right)\right)}^{\lambda -1}}{D_{i}\left(x,A\right)}, & \text{if}\;j\in A\cap H_{C}. \end{array}\right.$$

Evaluating (4) at $x={\tilde{c}}_{iS}$ gives the treatment choice probability $\mathrm{Pr}\left[j|A,{\tilde{c}}_{iS}\right]$ based on expected post‐surgery outcomes. Evaluating (4) at $x=c_{S}$ gives the hypothetical treatment choice probability $\mathrm{Pr}\left[j|A,c_{S}\right]$ if patient i knew the benchmark post‐surgery outcomes.

### 2.1 Online Experiment and Identification

Our online experiment addresses the limitations discussed in the previous section and facilitates the identification of the model parameters. As discussed in detail in Section 3, the design has two central features.

First, we randomize the allocation of information $I_{i}$ and elicit expected post‐surgery outcomes ${\tilde{c}}_{iS}\left(I_{i}\right)$ before patients make a treatment choice. By eliciting ${\tilde{c}}_{iS}\left(I_{i}\right)$, we eliminate this dimension of unobserved heterogeneity and can be agnostic about the belief distribution $B_{i}\left(\cdot |I_{i}\right)$. Randomization of $I_{i}$ alleviates endogeneity concerns about the way patients gather information. In addition, waiting times $w_{ij}$ are randomly assigned in the choice tasks, supporting the maintained exogeneity of waiting times with respect to unobserved determinants of treatment choice.

Second, we randomly assign patients either to the full set of treatments J or to the constrained set of treatments C, which excludes physiotherapy. In addition to implying exogeneity of A∈{J,C} with respect to $\varepsilon_{i}$, this random assignment helps identify the nesting parameter λ, because it creates exogenous variation in whether the set of available treatments contains one or two treatments from the conservative nest $H_{C}$. When physiotherapy is excluded, $A\cap H_{C}=\left\{G\right\}$ and λ drops out of the choice probabilities in (4), which reduce to multinomial‐logit probabilities over S and G. By contrast, when physiotherapy is included, $A\cap H_{C}=\left\{G,P\right\}$ and the substitution between surgery and the two conservative treatments depends on λ.

Given these identification considerations, we then estimate the nested logit model (4) evaluated at $x={\tilde{c}}_{iS}$ by maximum likelihood, where expected post‐surgery outcomes ${\tilde{c}}_{iS}\left(I_{i}\right)$ are elicited after the random allocation of $I_{i}$ and before any treatment choice is made.^3^

### 2.2 Patient Loss From Incorrect Beliefs

Consider the treatment choice probabilities in (4), both evaluated at the full set of treatments A=J. Denote by $j^{⋆}$ the chosen treatment from J, $j^{⋆}=\arg\;\max_{k\in J}\left\{EU_{ik}\right\}$. The only treatment for which benchmark and expected indirect utility can differ is surgery. We therefore define:

$$d_{iS}=U_{iS}-EU_{iS}=V_{iS}\left(c_{S}\right)-V_{iS}\left({\tilde{c}}_{iS}\right)=\beta^{\prime }\left(c_{S}-{\tilde{c}}_{iS}\right),$$

while $d_{ik}=0$ for k∈{G,P}. The term $d_{iS}$ is the difference between the utility surgery yields under the benchmark post‐surgery outcomes and the utility the patient expected when choosing. If $d_{iS}<0$, surgery is worse than expected in utility terms; if $d_{iS}>0$, it is better than expected. Whenever $c_{S}\neq {\tilde{c}}_{iS}$, patient i may make a treatment choice $j^{⋆}$ that differs from the choice they would have made knowing the benchmark post‐surgery outcomes $c_{S}$.

Then, expected patient surplus—net of the Euler constant—by choosing a treatment from J is (the derivations are in Appendix A, see also Train 2015):

(5) $${\tilde{PS}}_{ij^{⋆}}\left(J\right)=-\frac{1}{\alpha }\left(\ln D_{i}\left({\tilde{c}}_{iS},J\right)+d_{iS}\mathrm{Pr}\left[S|J,{\tilde{c}}_{iS}\right]\right),$$

where division by −α denotes that expected patient surplus is evaluated in terms of “units” of waiting time $w_{ij}$. Expected patient surplus has two components. The first term captures the surplus implied by the expected utility index the patient uses when choosing, while the probability‐weighted $d_{iS}$ term adjusts this surplus for the fact that benchmark post‐surgery outcomes may differ from expectations. Throughout, a tilde on a surplus or loss object indicates that treatment choices are made using expected post‐surgery outcomes ${\tilde{c}}_{iS}$; objects without a tilde are instead evaluated only at benchmark post‐surgery outcomes $c_{S}$.

Denote by $r^{⋆}$ the treatment associated with the highest benchmark indirect utility from J, $r^{⋆}=\arg\;\max_{k\in J}\left\{U_{ik}\right\}$, and by $U_{ir^{⋆}}=EU_{ir^{⋆}}+d_{ir^{⋆}}$ its benchmark indirect utility. This represents the best treatment the patient would choose if they knew the benchmark post‐surgery outcomes $c_{S}$. The associated patient surplus is:

$$PS_{ir^{⋆}}\left(J\right)=-\frac{1}{\alpha }\ln\;D_{i}\left(c_{S},J\right).$$

This is the benchmark surplus that would be obtained if patient i made a treatment choice based on the benchmark post‐surgery outcomes $c_{S}$ rather than the expected post‐surgery outcomes ${\tilde{c}}_{iS}$. Then, expected loss from incorrect beliefs by choosing a potentially inferior $j^{⋆}$ instead of the best treatment $r^{⋆}$ is:

(6) $$\Delta {\tilde{PL}}_{i}\left(J,J\right)=-\frac{1}{\alpha }\left(\ln\;D_{i}\left({\tilde{c}}_{iS},J\right)-\ln\;D_{i}\left(c_{S},J\right)+d_{iS}\mathrm{Pr}\left[S|J,{\tilde{c}}_{iS}\right]\right).$$

In the loss measures $\Delta PL_{i}\left(A_{1},A_{2}\right)$, the first argument $A_{1}$ is the set of treatments from which the patient actually chooses, and the second argument $A_{2}$ is the set whose best treatment under the benchmark post‐surgery outcomes $c_{S}$ defines the welfare reference.

### 2.3 Patient Loss From Limited Availability of Treatments

Here we illustrate how to measure the patient loss caused by limited availability of treatments, a situation in which patients correctly anticipate the benchmark post‐surgery outcomes $c_{S}$, so that $d_{iS}=0$, but physiotherapy (P) is not an available treatment. In other words, patients make choices from the constrained set of treatments C={S,G} rather than from the full set of treatments J={S,G,P}. In Section 3, we describe how we randomly assign patients to these two sets of treatments.

Denote by $r^{c}$ the treatment associated with the highest benchmark indirect utility from C, $r^{c}=\arg\;\max_{k\in C}\left\{U_{ik}\right\}$, and by $U_{ir^{c}}$ its benchmark indirect utility. Then, patient surplus under benchmark post‐surgery outcomes—net of the Euler constant—by choosing a treatment from C is:

(7) $$PS_{ir^{c}}\left(C\right)=-\frac{1}{\alpha }\ln\;D_{i}\left(c_{S},C\right).$$

As a consequence, the expected loss due to limited availability of treatments is:

(8) $$\Delta PL_{i}\left(C,J\right)\;=\;-\frac{1}{\alpha }\left(\ln\;D_{i}\left(c_{S},C\right)-\ln\;D_{i}\left(c_{S},J\right)\right).$$

The patient losses described in (6) and (8) are not mutually exclusive and can occur at the same time: patients may not correctly anticipate $c_{S}$ *and* may face a constrained set of treatments. In this case, formulae (6) and (8) can be combined to measure the total patient loss. Denote by $j^{c}$ the chosen treatment from C, $j^{c}=\arg\;\max_{k\in C}\left\{EU_{ik}\right\}$. Then, the total expected loss from (a) incorrect beliefs and (b) limited availability of treatments is:

(9) $$\begin{array}{c} \Delta {\tilde{PL}}_{i}\left(C,J\right)=\underset{\left(a\right)}{\underbrace{{\tilde{PS}}_{ij^{c}}\left(C\right)-PS_{ir^{c}}\left(C\right)}}+\underset{\left(b\right)}{\underbrace{PS_{ir^{c}}\left(C\right)-PS_{ir^{⋆}}\left(J\right)}}\\ =-\frac{1}{\alpha }\left(\ln\;D_{i}\left({\tilde{c}}_{iS},C\right)-\ln\;D_{i}\left(c_{S},J\right)+d_{iS}\mathrm{Pr}\left[S|C,{\tilde{c}}_{iS}\right]\right). \end{array}$$

## 3 Experimental Design

We designed an online choice experiment for a hip replacement procedure in the UK that enabled the evaluation of the welfare losses outlined in Section 2. Participants were independently randomised along two interventions in a 2×2 design, summarized in Table 1. In the information intervention, participants either received only the standard risk information typically provided during NHS surgical consultations $\left(I_{i}=0\right)$, or additionally received profile‐specific forecasts of post‐surgery outcomes for the clinical profile used in the experimental scenario, based on HES data $\left(I_{i}=1\right)$. In the treatment availability intervention, participants were assigned either a constrained set of two treatments—surgery and GP‐led management (C)—or the full set of treatments including also physiotherapy (J).

**TABLE 1:** Experimental design.

	2. Treatment availability intervention	
	Constrained set of treatments (C)	Full set of treatments (J)
	Surgery, GP‐led management	Surgery, GP‐led management, physiotherapy
1. Information intervention		
Baseline information $\left(I_{i}=0\right)$	Obs = 475	Obs = 481
Profile‐specific forecasts $\left(I_{i}=1\right)$	Obs = 474	Obs = 447

*Note:* All participants faced the same medical scenario and standard NHS information about surgical complications (Appendix Figures A1, A2, A3, A4 and Figure 2). The information intervention $\left(I_{i}=1\right)$ additionally provided profile‐specific forecasts of post‐surgery outcomes for the clinical profile described in the medical scenario. These forecasts covered extended hospital stay, hospital readmission within 6 months, and death within 90 days, presented as elevated relative to the average patient (Figure 3). The treatment availability intervention changed the set of treatments from the constrained set C to the full set J by adding physiotherapy with the opportunity to reassess surgery in 6 months (Figure 4).

The NHS does not systematically offer quantitative forecasts of post‐surgery outcomes across elective procedures when discussing surgical treatment with patients, and information provision is usually limited to general population risks. This is especially relevant for high‐risk patients, whose age and comorbidities may substantially alter expected post‐surgery outcomes relative to average patients (ISOS 2016; S. Shaw et al. 2020; S. E. Shaw et al. 2023; Vickery et al. 2025). In such settings, general risk information may be insufficient for patients to assess the expected value of surgery relative to non‐surgical management. The information intervention varied whether participants received only the standard NHS risk information or additional profile‐specific forecasts of post‐surgery outcomes for a high‐risk clinical profile, consistent with guidance that risk communication should account for individual clinical circumstances and use numerical information when available (General Medical Council 2020; National Institute for Health and Care Excellence 2021).

The treatment availability intervention targeted a complementary margin: when surgical risks are elevated, an active conservative pathway may become especially relevant. This margin is clinically relevant for hip replacement, which is commonly performed for osteoarthritis after other treatments have not worked, and for which exercise and physiotherapy are recognised non‐surgical treatments (NHS 2024; National Institute for Health and and Care Excellence 2022). It is also behaviourally relevant because prior work on high‐risk surgical decisions shows that non‐surgical alternatives may be perceived as inaction unless framed as active treatments (Ludwiczak et al. 2024).

**FIGURE 1:**
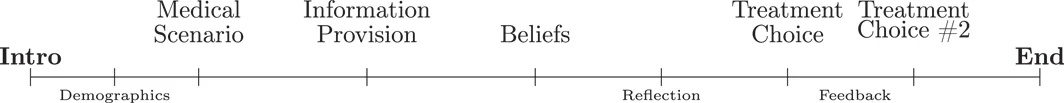
Choice experiment timeline.

We now describe the experiment timeline and the details of the interventions. Figure 1 summarises the main stages of the experiment. We began by describing the study background and eligibility criteria for participants. We explained to participants that the study aimed to understand how people use information and make decisions about major elective surgery. Participants were informed that they would answer a questionnaire from the point of view of a hypothetical patient who had a condition that could be treated by surgery and some pre‐existing health problems. They were told that they would receive details about this patient's background and were asked to imagine themselves as that person when making decisions. Because the online experiment was run in May 2021, participants were instructed to imagine a post‐COVID setting in which surgery and medical procedures were conducted safely and efficiently, so that the clinical scenario remained realistic and unaffected by pandemic‐related concerns. We mentioned that the questionnaire would typically take less than 25 minutes, but they had up to 90 minutes to complete it.Participants had to be residents of the United Kingdom, aged 50 or older, and not be awaiting any major surgery. We did not inform participants about the randomization process at any point. After participants agreed to take part in the study, we collected general demographic information and asked questions about aspects of their health status and life.

All medical scenarios described in the experiment were developed in collaboration with clinical researchers experienced in perioperative medicine and critical care as part of an ongoing research program on shared decision‐making for high‐risk surgery. In the second main section of the experiment, we presented participants with the hypothetical medical scenario. We described a patient called “Sam.” Sam was described as a 76‐year‐old man or woman, matching the self‐reported gender of the participant, with multiple comorbidities that placed them at elevated risk of post‐surgery complications. Sam's pre‐existing conditions included cardiovascular disease, diabetes, chronic kidney disease, and depression. Participants were asked to imagine being Sam and to make decisions as if they were in Sam's position, while retaining their own background, beliefs, and values.

**FIGURE A1:**
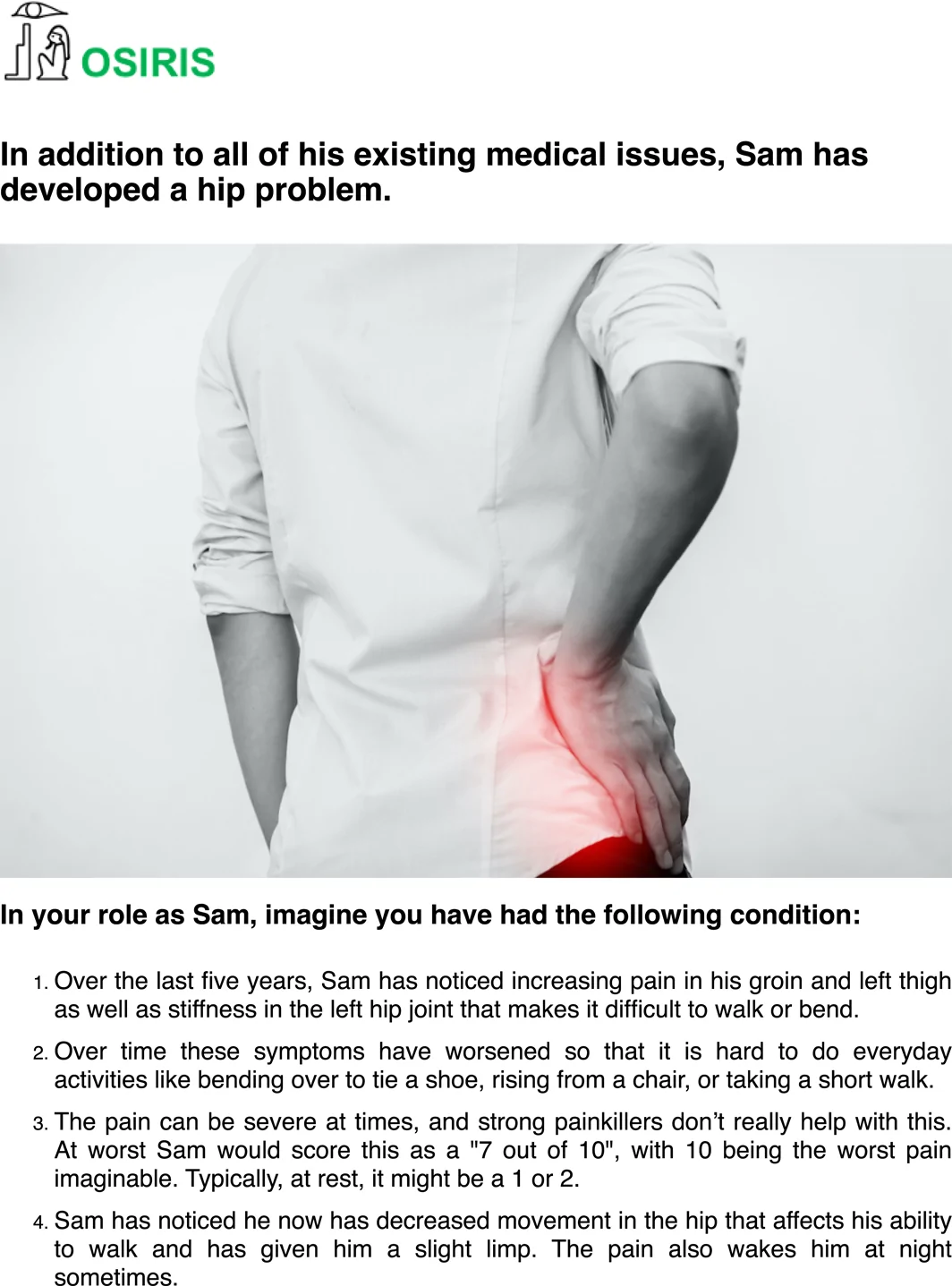
Medical scenario—Condition.

Participants were then provided with information about Sam's medical condition (Appendix Figure A1). In particular, it was explained that over the past 5 years, Sam had experienced increasing pain in their groin and left thigh, as well as stiffness in the hip joint that made it difficult to walk or bend. These symptoms had progressively worsened, limiting everyday activities such as bending to tie a shoe, rising from a chair, or taking a short walk. The pain could be severe at times, rated 7 out of 10 at worst, though typically 1 or 2 at rest. Sam had also noticed decreased movement in the hip, which had caused a slight limp and disturbed their sleep.

**FIGURE A2:**
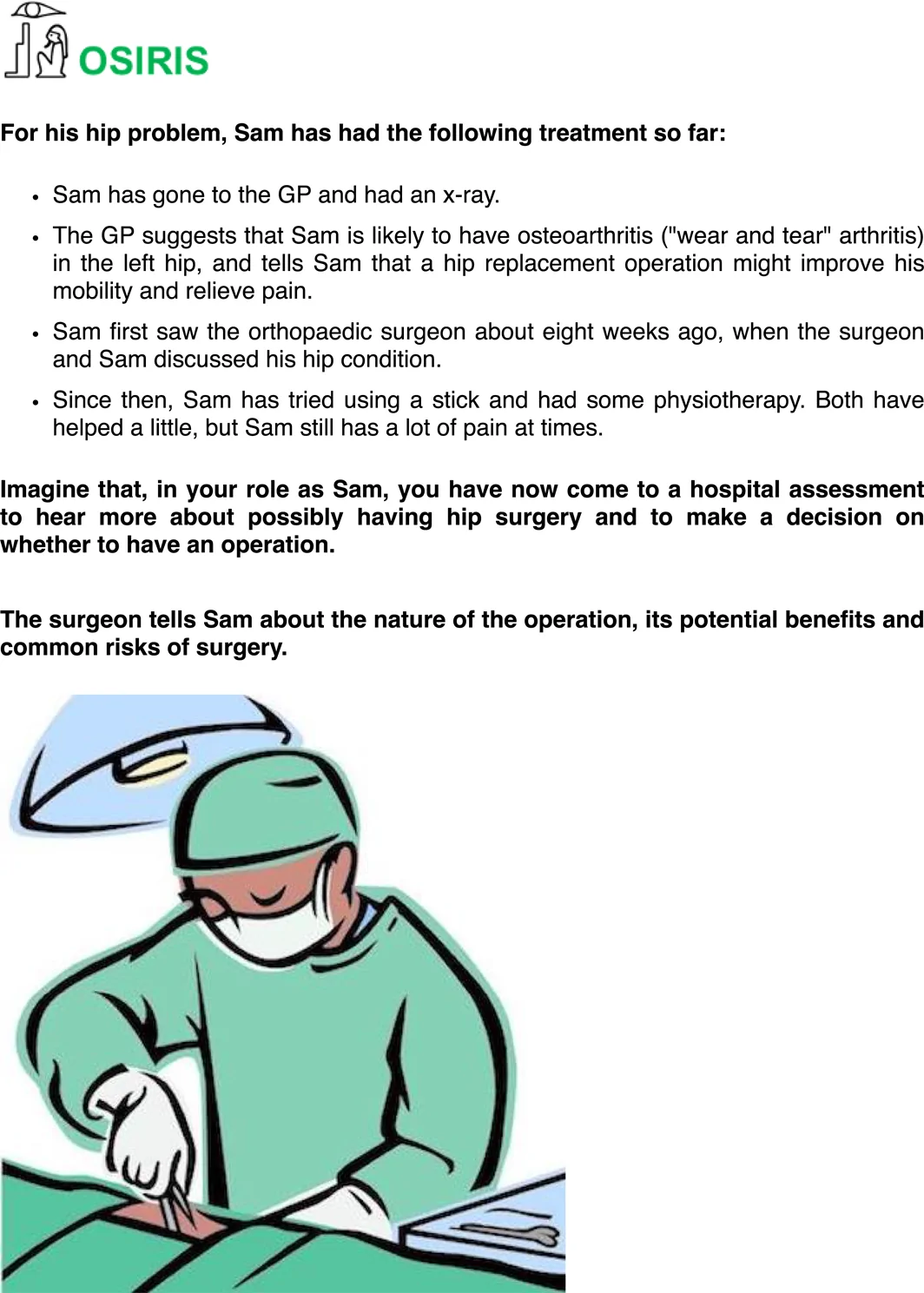
Medical scenario—Management of hip problem.

Sam's GP had referred them to an orthopedic surgeon to discuss whether hip replacement surgery might improve their mobility and relieve pain. Prior to the surgical consultation, Sam had tried conservative treatments, including some physiotherapy, which had helped a little but had not resolved the pain (Appendix Figure A2).

**FIGURE 2:**
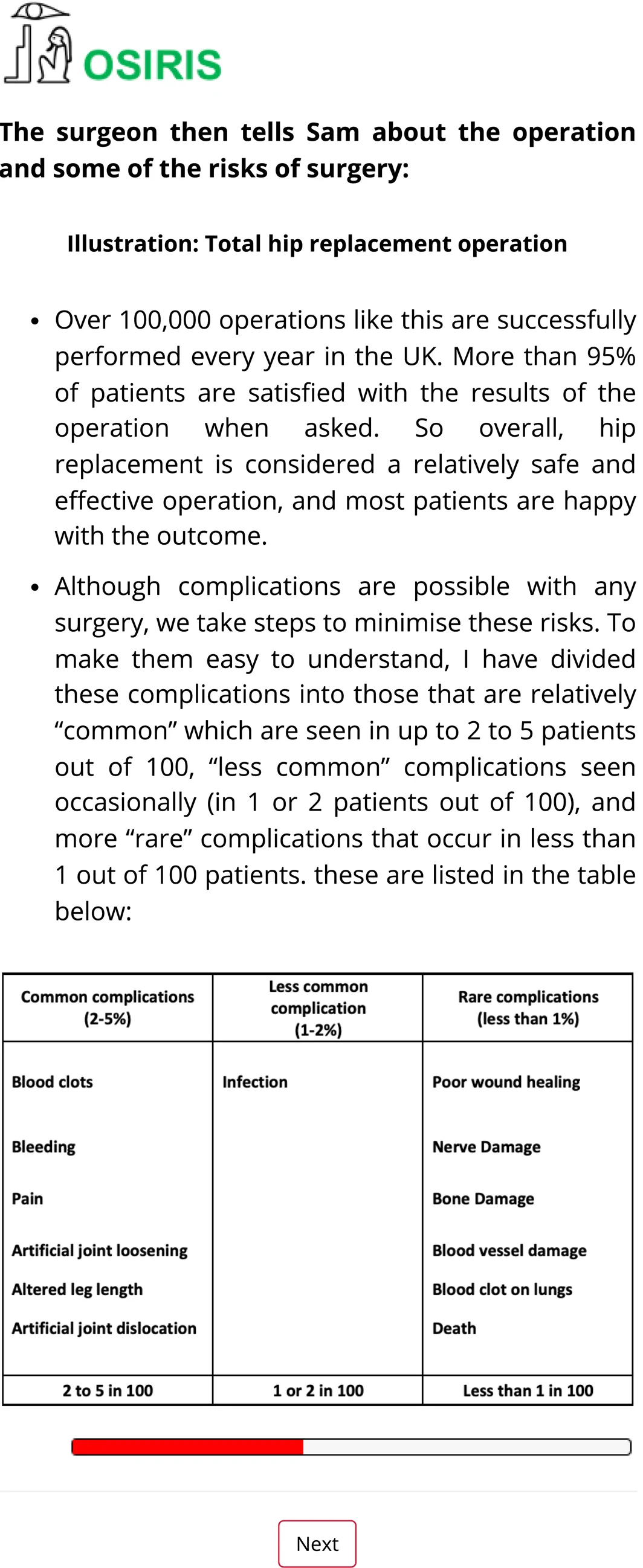
Medical scenario — Risks of surgery.

**FIGURE A3:**
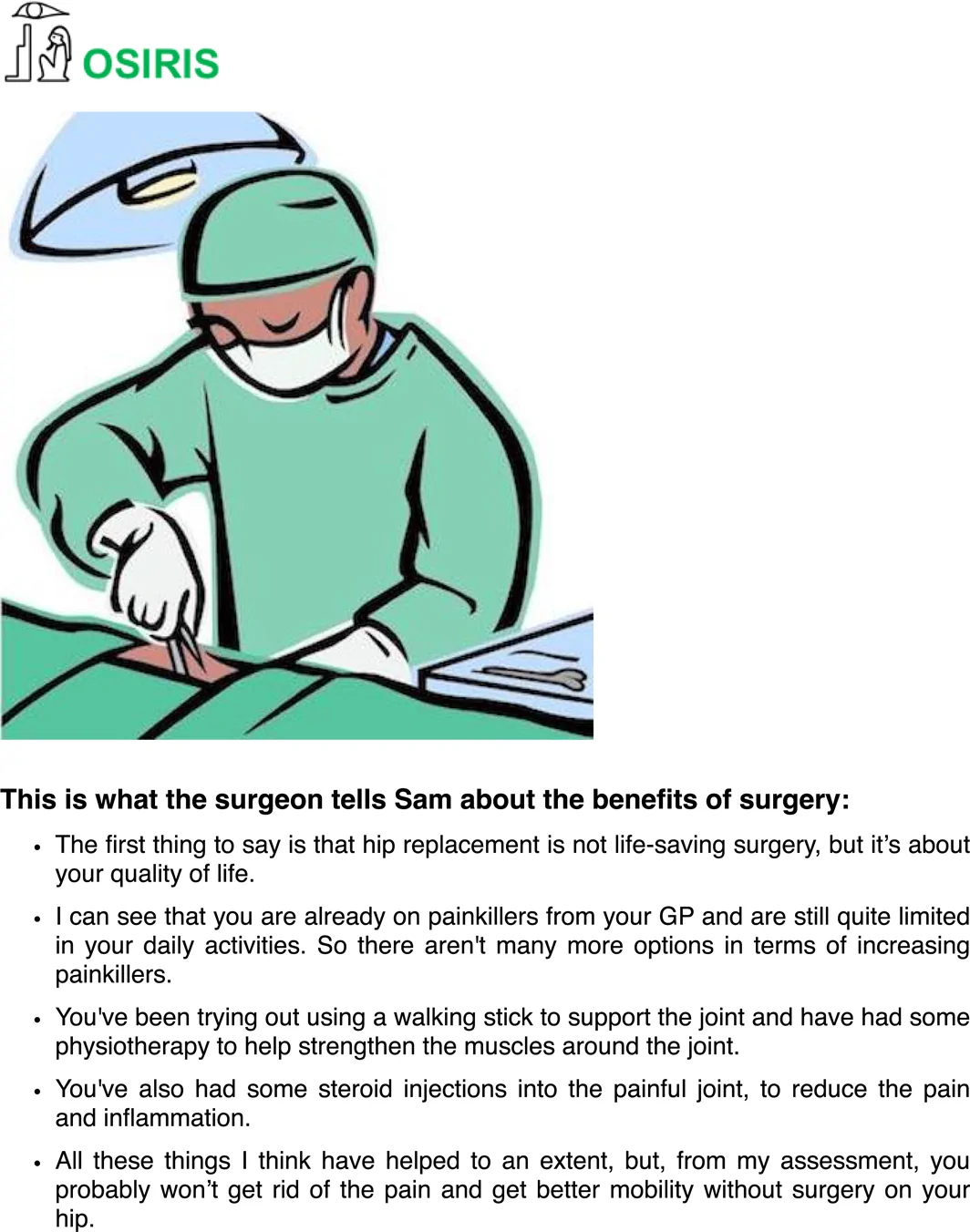
Medical scenario—Expected benefits of surgery.

**FIGURE A4:**
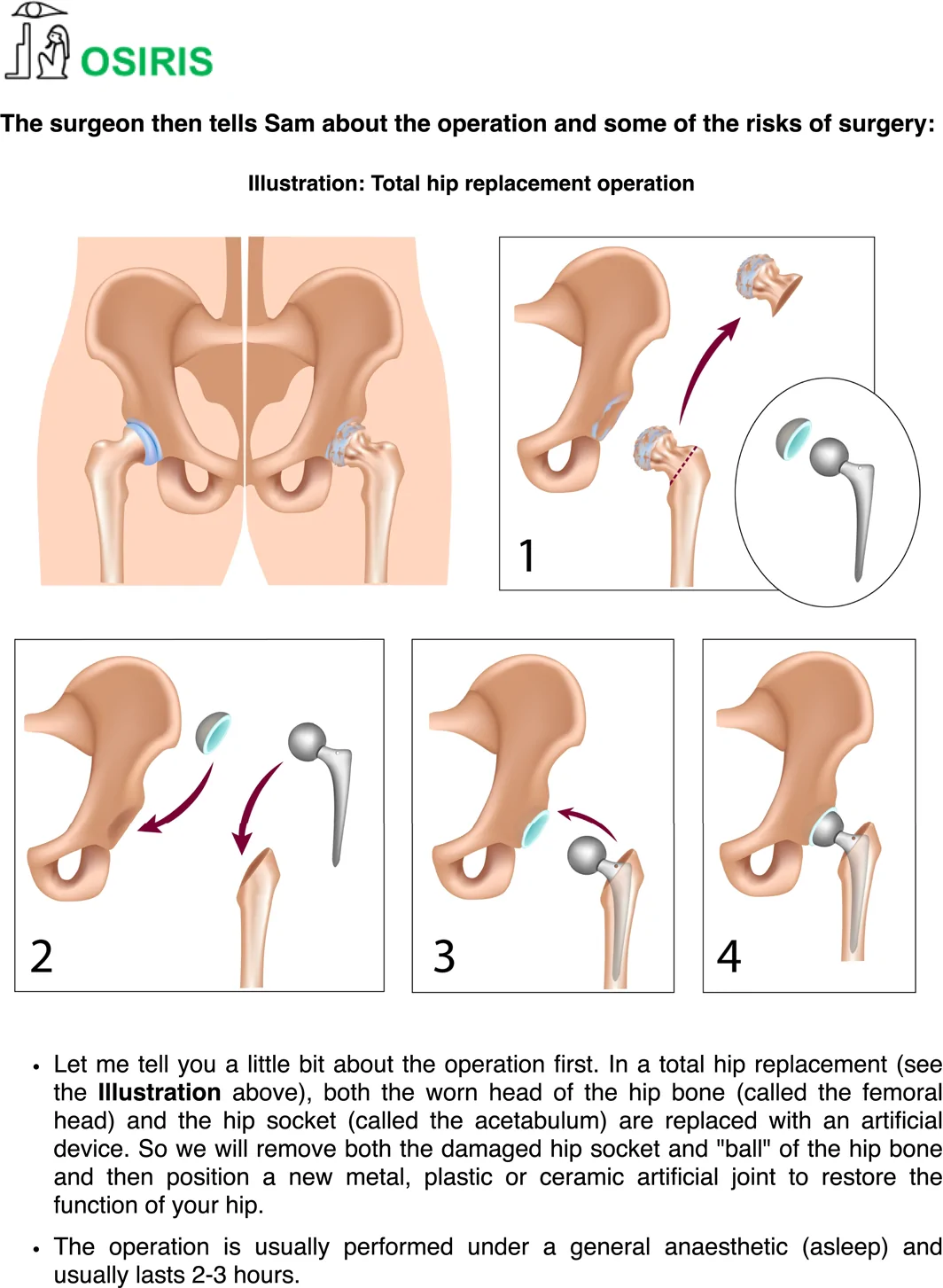
Medical scenario—Hip replacement.

During the simulated consultation, the surgeon explained the nature of the hip replacement procedure, its potential benefits, and common surgical risks. This information described potential complications in general terms and included reassuring language consistent with standard NHS practice for this procedure (e.g., that hip replacement is “considered a relatively safe and effective operation” and that “steps are taken to minimize these risks”). All participants received this standard NHS information about surgical complications, categorized by frequency (common, less common, and rare), as shown in Figure 2 (see also Appendix Figures A3 and A4 for the expected benefits and the hip replacement procedure).

It is important to note that Sam's medical history included prior experience with conservative treatments, such as having had some physiotherapy in the past. This does not conflict with the treatment availability intervention, because the intervention captured a distinct decision constraint at the point of the surgical decision. In the orthopedic pathway described by S. Shaw et al. (2020), conservative management may occur before referral, but the subsequent decision‐making process is organized around whether to proceed with surgery. More broadly, S. Shaw et al. (2020) emphasize that high‐risk surgical decisions can be shaped by surgical momentum and by communication practices that frame the patient's condition in relation to a surgical solution. Our treatment availability intervention therefore evaluated the importance of explicitly including physiotherapy as an active treatment alternative at the point at which participants decided whether to proceed with surgery.

**FIGURE 3:**
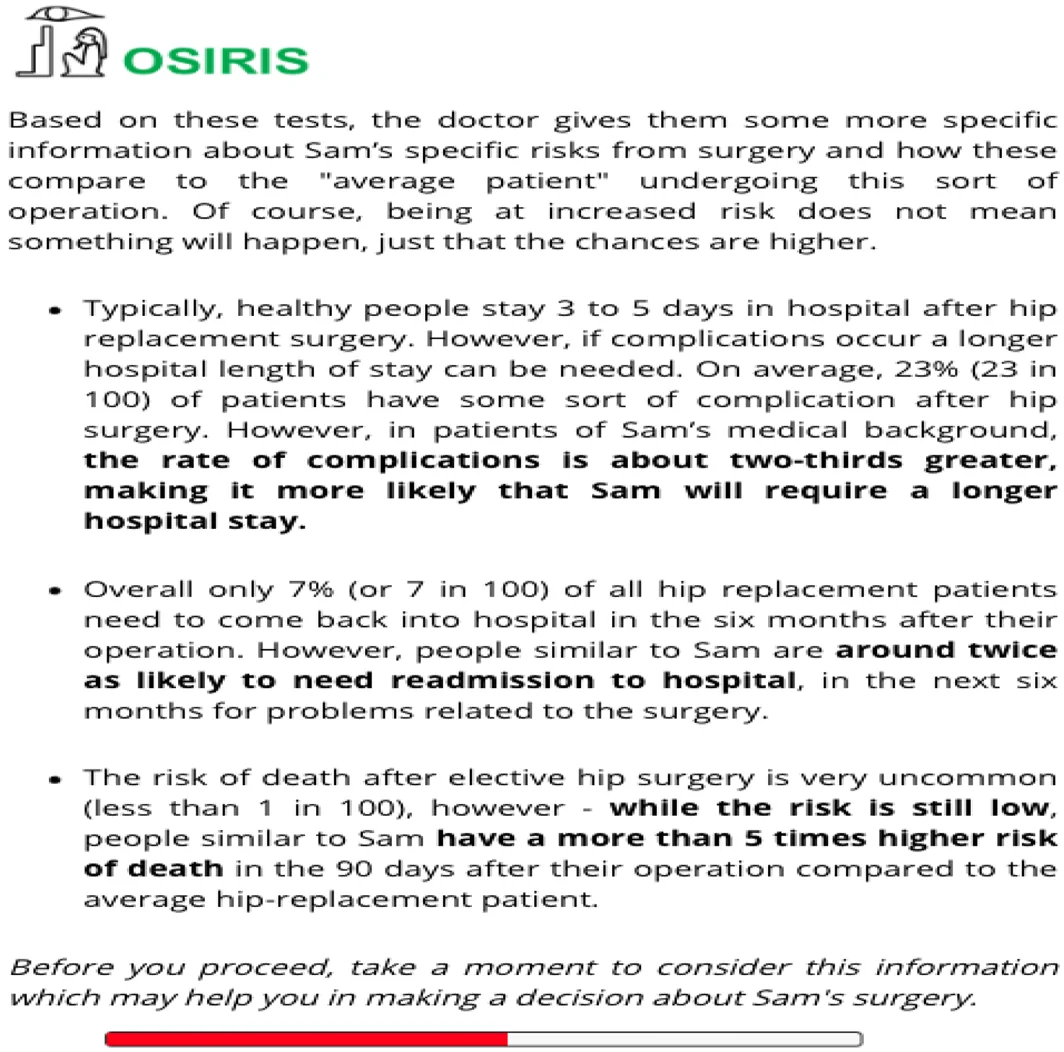
Information intervention.

After participants received the standard NHS background information, we implemented the information intervention. Participants assigned to the control arm of the intervention $\left(I_{i}=0\right)$ made their decisions based on the general risk information provided during the simulated surgical consultation described so far (Figure 2). Participants assigned to the information intervention $\left(I_{i}=1\right)$ received, in addition to the same baseline information, average‐patient probabilities for three post‐surgery outcomes, together with profile‐specific deviations from these averages computed from HES data for Sam's clinical profile. Specifically, the information intervention reported average‐patient probabilities for extended hospital stay, hospital readmission within 6 months, and death within 90 days, and then stated the corresponding relative deviations for Sam's clinical profile (Figure 3).^4^

We computed the *benchmark* values $c_{S}$—see Section 2—as the predicted probabilities of the three post‐surgery outcomes based on HES data for patients with Sam's characteristics. We then used $c_{S}$ as a reference against which to compare participants' beliefs about Sam's post‐surgery outcomes. Importantly, the information intervention used these benchmark post‐surgery outcomes to construct the profile‐specific deviations shown to participants, but it did not directly display $c_{S}$.

Before making a treatment choice, we asked participants to report their expected values of the three post‐surgery outcomes: the probabilities of extended hospital stay, hospital readmission within 6 months, and death within 90 days. Following the notation in Section 2, these *elicited* values correspond to the relevant components of ${\tilde{c}}_{iS}\left(I_{i}\right)$, and they explicitly depend on the random allocation of information $I_{i}\in \left\{0,1\right\}$. We elicited beliefs on a scale from 0 to 100: “Of 100 patients similar to the one described, how many stay in the hospital longer than the average time?” We used a similar format for hospital readmission within six months and death within 90 days.

After eliciting beliefs, we introduced a reflection section between the presentation of available treatments and the treatment choice. For instance, participants answered questions about whom they would ask for support or advice in making this treatment choice, and how easy they found it to make this treatment choice. The reflection section was meant to allow participants to evaluate the treatment‐choice problem more thoroughly. In the next survey section, participants chose their preferred treatment from the available set of treatments.

**FIGURE 4:**
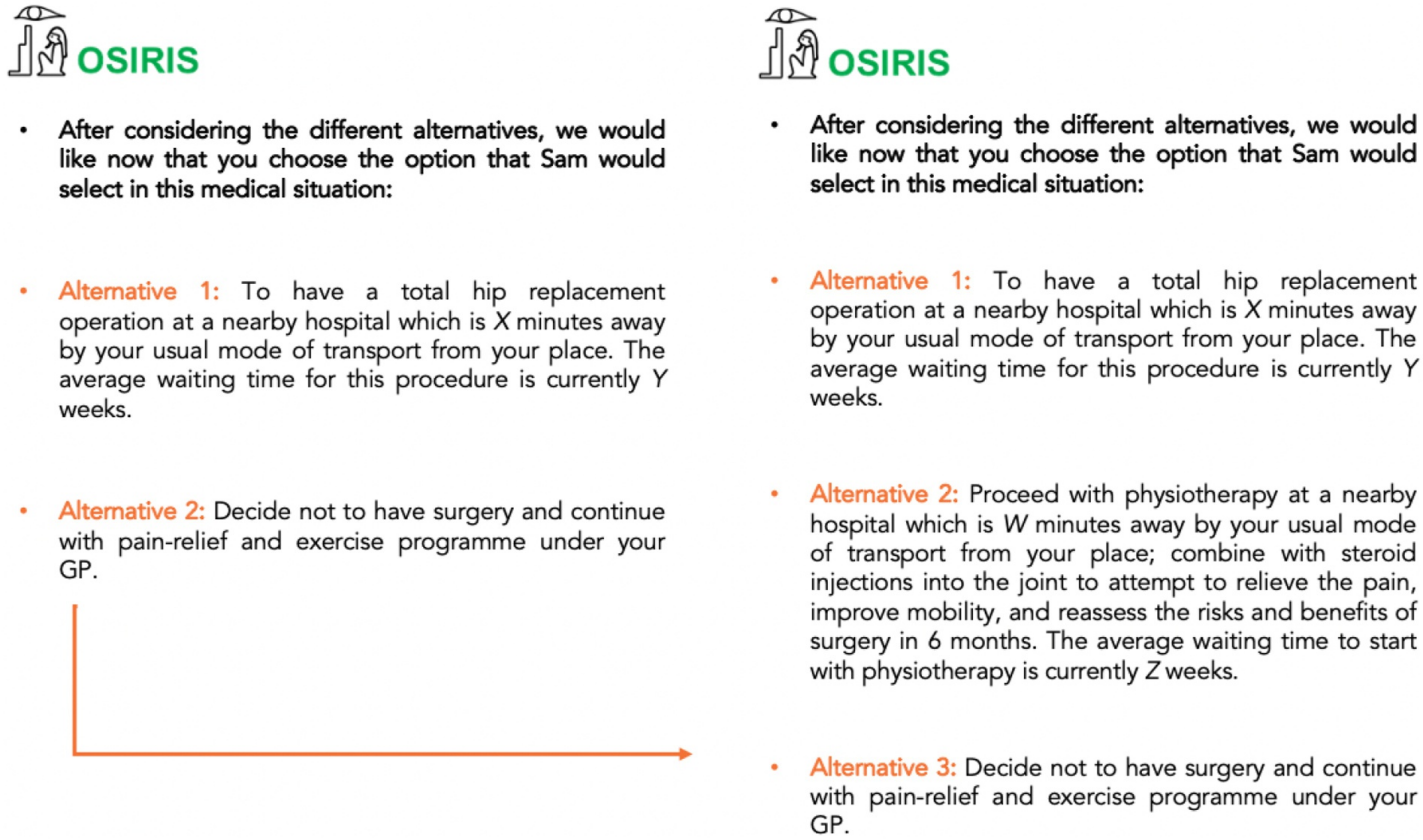
Treatment availability intervention.

Participants randomly allocated to the constrained set of treatments, C, decided whether to (i) undergo hip replacement or (ii) continue with pain relief and an exercise program under their GP. Participants randomly allocated to the full set of treatments, J, decided among (i), (ii), and (iii) physiotherapy with the opportunity to reassess surgery in 6 months (Figure 4).

As described in Section 2, in our analysis we use waiting time, denoted by $w_{ij}$, as the cost numeraire in our welfare calculations. We randomised at the participant level the waiting time associated with surgery and physiotherapy, while we set $w_{iG}=0$ for all participants (where G denotes GP‐led management). In contrast to the post‐surgery outcomes, waiting times were observed directly by participants when making a treatment choice, and we did not elicit beliefs about them. This experimental design choice is twofold. First, randomising waiting times ensures the maintained exogeneity assumption discussed in Section 2. Second, including waiting times in the analysis reflects the empirical reality that waiting time is a key determinant of treatment choice (Burge et al. 2004; Windmeijer et al. 2005; Sivey 2012). In the context of the NHS, waiting times function as a price of care that rationalises demand between alternative hospitals (Sivey 2012, 2018). Recent work on hip replacement has shown that waiting times vary substantially across providers and that patients with worse pre‐surgery health tend to wait less, reflecting implicit prioritisation (Kasteridis et al. 2023). Waiting times were drawn from a uniform distribution between 1 and 21 weeks, reflecting realistic variation in NHS elective care.^5^

In the final section of the survey, we presented to all participants the benchmark post‐surgery outcomes $c_{S}$. As shown in Appendix Figure A6, this final feedback displayed the benchmark values for Sam's clinical profile directly. Finally, we asked participants to make the same treatment choice again to examine whether, once everyone faced the same information about the benchmark post‐surgery outcomes, participants deviated from their original choices and updated them based on the more accurate information provided at that stage.

## 4 Data

Our target demographic group included UK residents aged 50 or older who were not scheduled for any major surgical procedure. As part of the broader OSIRIS Research Programme, our study focuses on high‐risk patients for whom the outcomes of a surgery are substantially more uncertain. On the one hand, providing more precise information about post‐surgery outcomes may reduce misalignment between expected and experienced complications for patients with less certain outcomes (i.e., where misalignment may be larger). On the other hand, stressing the availability of a less invasive treatment such as physiotherapy is a relatively low‐cost intervention that could greatly benefit some of these high‐risk patients, especially those more likely to face serious post‐surgery complications.

**FIGURE A5:**
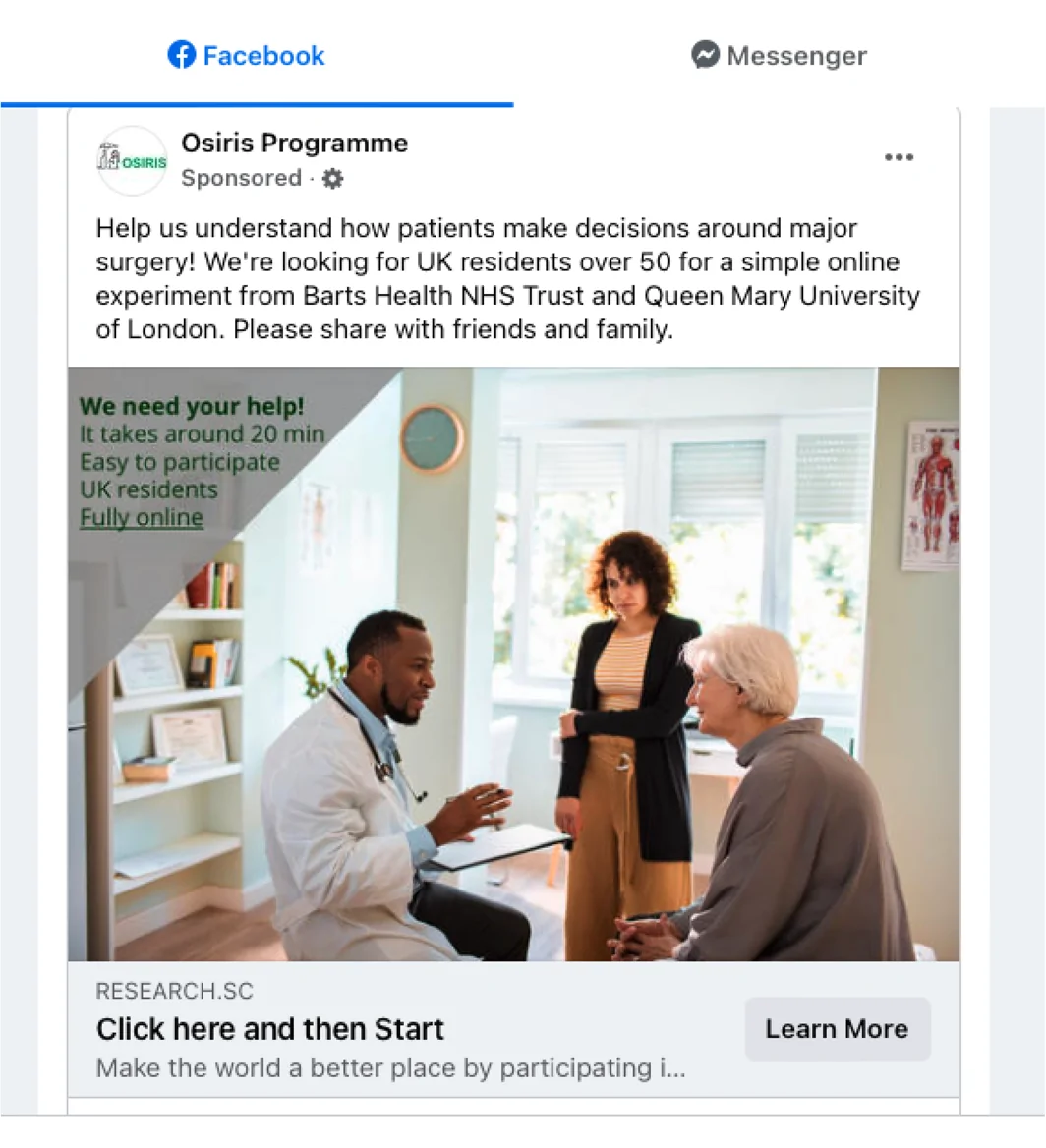
Facebook (meta) ad.

**FIGURE A6:**
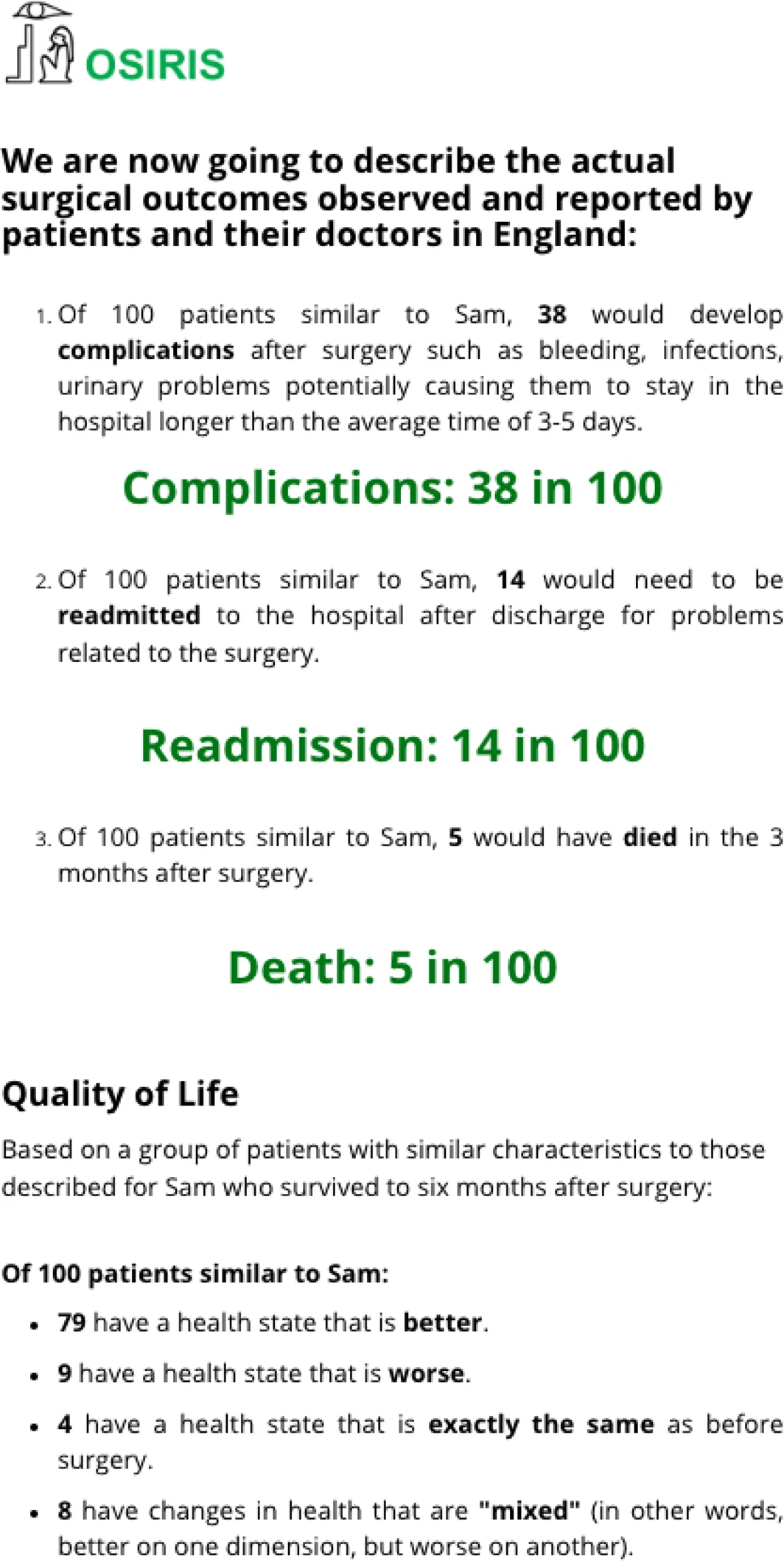
Final feedback on benchmark post‐surgery outcomes.

We recruited participants through the OSIRIS Programme's partner charities^6^ (e.g., social media platforms and mailing lists) and Facebook ads. Appendix Figure A5 shows the poster design used to invite participants. The online experiment was run in May 2021. Approximately 75% of the responses were obtained through Facebook. The final sample contains 1877 observations after applying two quality filters to the data.^7^ First, we excluded from the main analysis five participants whose reported age group was under 50. Second, we excluded 192 participants with relatively fast completion times (less than 10 minutes) to reduce the chance of low‐quality responses. We do not observe substantial changes to our results when considering the entire sample.

Table 2 shows descriptive statistics from the final sample. Overall, the age bands are well distributed above 50 and approximate the population likely to need a hip replacement within a reasonable number of years, with most respondents aged 60–70 years. For instance, 41% of our sample reported suffering from arthritis, one of the main conditions that can cause substantial hip joint damage. Also consistent with the age distribution, 71% of our sample reported being retired. The sample contains relatively more women (86%) than men, and education levels are fairly spread between low (less than an undergraduate degree), medium (undergraduate degree), and high (postgraduate degree). 93% of respondents are white British, which is somewhat consistent with the UK's white population (81%).

**TABLE 2:** Descriptive statistics.

Variable	Categories	Frequency (%)	Missings (%)
Age group	50–59	294 (15.7%)	1 (0.05%)
	60–69	770 (41.0%)	
	70–79	718 (38.3%)	
	80+	94 (5.0%)	
Female	0	270 (14.4%)	0 (0.0%)
	1	1607 (85.6%)	
White British	0	131 (7.0%)	0 (0.0%)
	1	1746 (93.0%)	
Employment	Other	151 (8.0%)	0 (0.0%)
	Retired	1328 (70.8%)	
	Working	398 (21.2%)	
Education level	Low	835 (44.5%)	0 (0.0%)
	Medium	412 (21.9%)	
	High	630 (33.6%)	
Arthritis	0	1107 (59.0%)	0 (0.0%)
	1	770 (41.0%)	
Obs		1877 (100.0%)	

*Note:* Education levels are defined as low (less than an undergraduate degree), medium (undergraduate degree), and high (postgraduate degree).

Appendix Table A1 shows the balance test of baseline characteristics by experimental intervention group. All *p*‐values are well above 10%, and we grouped age bands into ages below and above 65, given that we have few observations in the 50s band.

## 5 Results

Following the experimental timeline, we first discuss the effect of the information intervention on beliefs about post‐surgery outcomes. We then present the results of the discrete choice experiment along with the welfare analysis for the model of incorrect beliefs and limited availability of treatments presented in Section 2.

### 5.1 Accuracy of Beliefs and Information Intervention

To explore the accuracy of participants' beliefs about the three post‐surgery outcomes, we compare elicited beliefs ${\tilde{c}}_{iS}$ against benchmark probabilities $c_{S}$ derived from HES. HES data provide comprehensive administrative records of hospital activity in England, from which we compute benchmark probabilities for the three post‐surgery outcomes for Sam's clinical profile.

Table 3 shows how beliefs for the three post‐surgery outcomes differ for the control $\left(I_{i}=0\right)$ and intervention $\left(I_{i}=1\right)$ groups. We estimate the following regression:

(10) $$Y_{i}^{\text{Dev}}=\gamma_{0}+\gamma_{1}{\text{Intervention}}_{i}+u_{i},$$

where $Y_{i}^{\text{Dev}}$ is the difference, for each post‐surgery outcome, between the elicited belief and the benchmark value, while ${\text{Intervention}}_{i}$ is an indicator for the random allocation of information $I_{i}=1$.

**TABLE 3:** Beliefs and information intervention.

	${Stay}^{\text{Dev}}$	${Readmission}^{\text{Dev}}$	${Death}^{\text{Dev}}$
	(1)	(2)	(3)
Intervention	$6.190^{***}$	$8.226^{***}$	$1.311^{***}$
	(0.928)	(0.763)	(0.506)
Constant	‐$10.006^{***}$	$4.183^{***}$	$3.982^{***}$
	(0.675)	(0.508)	(0.370)
$R^{2}$	0.023	0.059	0.004
Obs	1877	1877	1877

*Note:* Dependent variables are deviations between expected and benchmark post‐surgery outcomes, defined as elicited beliefs minus the corresponding benchmark probabilities. Both elicited and benchmark probabilities are measured on a 0‐100 scale. Robust standard errors in parentheses. *p<0.1; **p<0.05; ***p<0.01.

The estimates in Table 3 show that both control $\left(\gamma_{0}\right)$ and intervention $\left(\gamma_{0}+\gamma_{1}\right)$ groups, on average, understate the probability of extended hospital stay (Column (1)), while they overstate the probabilities of hospital readmission (Column (2)) and of death (Column (3)). The main finding is that the intervention group reports higher average values for all outcomes relative to the control group, indicating that the information intervention systematically shifts beliefs upward.

Importantly, this upward shift has different implications for belief accuracy depending on the outcome. For extended hospital stay, where the control group underestimates the benchmark probability by 10 percentage points (p.p.) on average, the intervention effect of 6.19 p.p. moves treated participants closer to the benchmark, reducing their average misalignment to approximately four p.p.

In contrast, for hospital readmission within 6 months and death within 90 days, where the control group already overestimates the benchmark probabilities, the intervention amplifies this overestimation, pushing the beliefs of the intervention group further away from the benchmark value. The results in Table 3 are robust to the inclusion of demographics. Appendix Table A2 presents specifications that additionally control for age group, gender, ethnicity, employment status, education level, and arthritis. The coefficient on the intervention indicator remains statistically significant and virtually unchanged in magnitude across all three outcomes. Notably, higher education is associated with smaller belief deviations for hospital readmission and death, suggesting that more educated participants hold beliefs closer to the benchmark predictions for these two outcomes regardless of intervention assignment.

**FIGURE 5:**
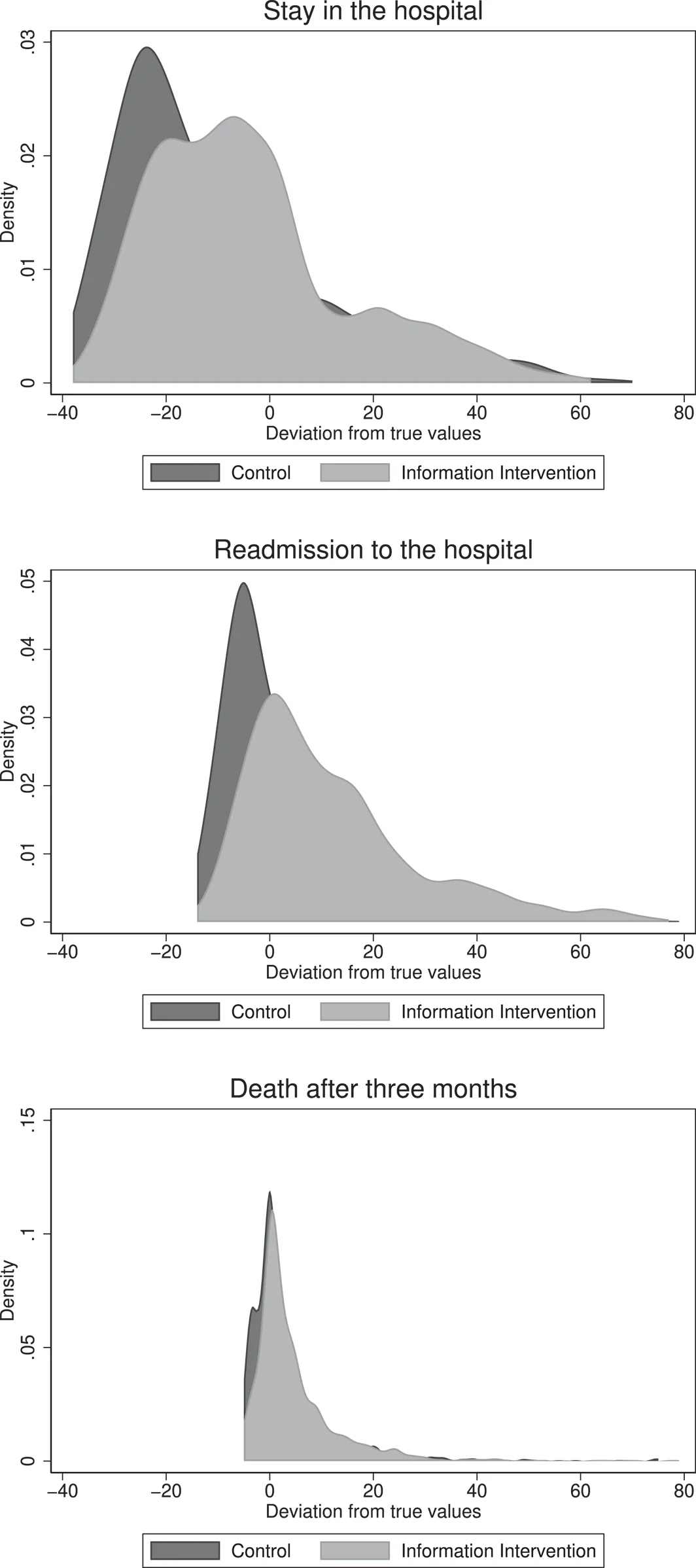
Accuracy of beliefs and information intervention.

To explore the heterogeneity of these effects, Figure 5 plots kernel density estimates for the three post‐surgery outcomes by intervention group status. In general, the densities for the intervention group are consistently shifted to the right of the control densities. A key result when assessing the distribution of beliefs is that, although this shift moves the intervention group closer to the benchmark prediction in some cases (i.e., peaks of the densities closer to zero), it also causes some participants to overestimate post‐surgery outcomes more substantially (thick tails to the right of the densities).

### 5.2 Discrete Choice Experiment and Welfare Estimates

This section analyses participants' treatment choices, how these choices vary with our interventions, and the consequent welfare effects evaluated according to the model introduced in Section 2.

As described in Section 3, we randomly allocated participants to one of two sets of treatments: the full set J={S,G,P}, including physiotherapy (P), and the constrained set C={S,G}, excluding it. Participants allocated to the constrained set C decided whether to undergo hip replacement surgery or to continue with a non‐invasive exercise program along with painkillers under the supervision of the GP. Because surgery is typically the default treatment for a patient with a hip condition like Sam, a possible response is to highlight, during the consultations leading to the surgical decision, the availability of additional recognised non‐surgical treatments, such as physiotherapy (NHS 2024; National Institute for Health and and Care Excellence 2022).

**FIGURE 6:**
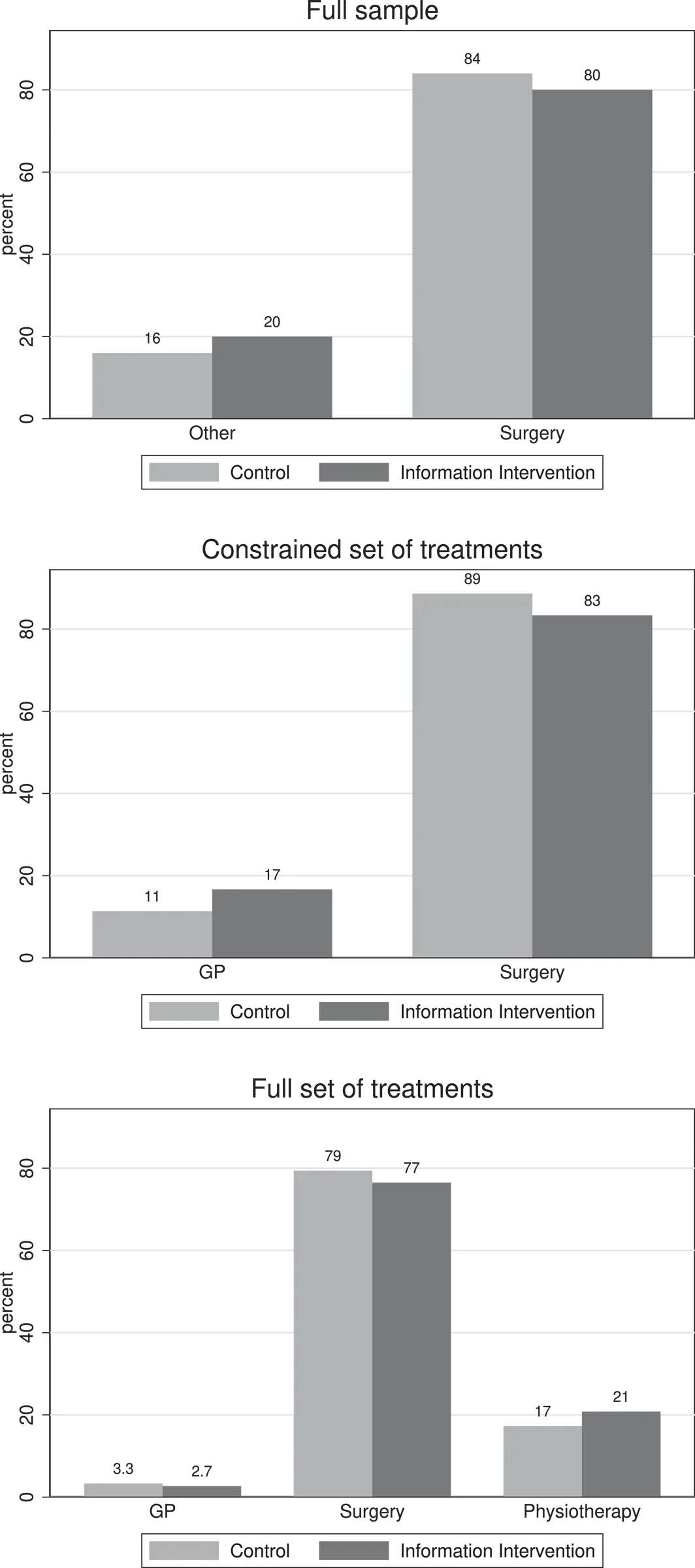
Treatment choices by intervention.

Figure 6 summarises treatment choices across experimental groups. The top plot shows the likelihood of choosing surgery rather than another treatment for the entire sample. Surgery is chosen by a large majority of participants across all experimental groups. This result is consistent with observed decisions and experimental evidence from patients and clinicians (Ludwiczak et al. 2024).

In the full sample, participants assigned to the information intervention are four p.p. Less likely to choose surgery, and the difference is statistically significant (Pearson $\chi^{2}\left(1\right)$ = 5.029, *p*‐value = 0.025). This is consistent with the information intervention leading participants to perceive a higher likelihood of poor post‐surgery outcomes.

We see similar results when examining the groups assigned to different sets of treatments separately. The middle plot shows the choices of the group randomly allocated to the constrained set of treatments C. The probability of choosing surgery falls from 89% to 83%. The six p.p. Difference is also statistically significant for this group (Pearson $\chi^{2}\left(1\right)$ = 5.526, *p*‐value = 0.019).

The bottom plot displays choices for participants assigned to the full set of treatments J, which additionally includes physiotherapy. In this group, surgery remains the most frequently chosen treatment, but at lower levels than in the constrained set C: 79% of participants in the control group and 77% in the intervention group choose surgery. Physiotherapy attracts a sizable share of choices, increasing from 17% in the control group to 21% in the intervention group. By contrast, GP‐led management is chosen by only 3.3% and 2.7% of participants in the control and intervention groups, respectively. The overall distribution of choices does not differ significantly across information groups within J (Pearson $\chi^{2}\left(2\right)$ = 2.106, *p*‐value = 0.349).

Taken together, Figure 6 shows two distinct patterns. First, the information intervention shifts choices away from surgery, but this effect is most visible when physiotherapy is not available: surgery falls by six p.p. in the constrained set C, compared with two p.p. in the full set J. Second, adding physiotherapy substantially changes the composition of non‐surgical choices. When physiotherapy is available, GP‐led management becomes rare, while physiotherapy attracts a much larger share of participants. This pattern suggests that physiotherapy substitutes especially for GP‐led management, consistent with modeling the two non‐surgical treatments as belonging to the same conservative nest, $H_{C}=\left\{G,P\right\}$ (see Section 2).

As described in Section 3, participants were presented with a final opportunity to revise their treatment choice after receiving feedback on predicted post‐surgery outcomes at the end of the survey. We do not focus on these revised choices in our analysis for several reasons. First, the switching rates were low, with only 66 participants in the C group and 92 participants in the J group who revised their initial choice. This limited variability constrains our ability to draw inferences about the determinants of choice revision. Second, our static discrete choice model is primarily designed to evaluate decisions under naturally occurring belief heterogeneity—the policy‐relevant scenario for understanding how patients make choices in clinical settings—but is not equipped to appropriately handle the ramifications of learning and/or regret.

#### 5.2.1 Choice Model and Welfare Estimates

We estimate the nested logit model presented in Section 2 using participants' elicited beliefs ${\tilde{c}}_{iS}$ for extended hospital stay, hospital readmission, and death. The nest structure groups the two non‐surgical treatments, GP‐led management and physiotherapy, into a single conservative nest, leaving surgery as a singleton invasive treatment. This structure, in line with the evidence from Figure 6, allows the unobserved components of utility to be correlated within the conservative nest, so that the two non‐surgical treatments act as closer substitutes for one another than for surgery. The nesting parameter λ governs the degree of this within‐nest correlation.

Table 4 shows the estimation results of the nested logit model (Panel A) and the implied patient loss measures from incorrect beliefs about post‐surgery outcomes and limited availability of treatments (Panel B).^8^ Panel A indicates that, holding the expected post‐surgery outcomes fixed, participants exhibit a strong baseline preference for surgery (the treatment whose constant $\delta_{S}$ is normalized to zero) over both GP‐led management (coefficient = −2.433, p<0.001) and physiotherapy (coefficient = −1.781, p<0.001). The coefficients on the expected post‐surgery outcomes carry the expected signs: beliefs about hospital readmission within 6 months (−0.017, p<0.001) and death within 90 days (−0.016, p=0.008) both reduce the likelihood of choosing surgery, while the coefficient on expected extended hospital stay is statistically insignificant (0.001, p=0.73). In other words, participants who expect a higher probability of hospital readmission or death are less likely to choose surgery.

**TABLE 4:** Nested logit and patient loss estimates.

Panel A: Model estimates (Obs = 1877)						
			Expected post‐surgery outcomes $\left({\tilde{c}}_{iS}\right)$		
	GP $\left(\delta_{G}\right)$	Physio $\left(\delta_{P}\right)$	Waiting (α)	Stay	Readmission	Death
Estimate	−2.433***	−1.781***	−0.017***	0.001	−0.017***	−0.016***
SE	(0.156)	(0.114)	(0.006)	(0.004)	(0.005)	(0.006)
Nesting parameter λ (nests $H_{C}=\left\{G,P\right\}$ and $H_{S}=\left\{S\right\}$): 0.245*** (0.063).						
Log‐likelihood =−925.80; McFadden $R^{2}=0.171$; LR test versus multinomial logit: $\chi^{2}\left(1\right)=60.11$, p<0.001						

Panel B: Patient loss estimates						
	Obs	Mean	Std. Dev.	[95% CI low	95% CI upper]	
(a) Expected loss from incorrect beliefs about post‐surgery outcomes						
$\Delta {\tilde{PL}}_{i}\left(J,J\right)$	928	−1.12	3.07	−3.61	−0.49	
$\Delta {\tilde{PL}}_{i}\left(C,C\right)$	928	−0.89	2.62	−2.87	−0.37	
(b) Expected loss from limited availability of treatments						
$\Delta PL_{i}\left(C,J\right)$	928	−4.13	1.03	−11.25	−2.06	
Total expected loss: (a) + (b)						
$\Delta {\tilde{PL}}_{i}\left(C,J\right)$	928	−5.02	2.87	−13.55	−2.72	

*Note:* Panel A presents the estimated coefficients for the nested logit model described in Section 2. The nests are $H_{C}=\left\{G,P\right\}$ for the conservative non‐surgical treatments and $H_{S}=\left\{S\right\}$ for surgery. Panel B presents: (a) the patient loss from incorrect beliefs about post‐surgery outcomes, (b) the patient loss from limited availability of treatments (the exclusion of physiotherapy from the constrained set C), and the total expected patient loss, all evaluated on the subsample assigned to the full set of treatments J (Obs = 928) for which both (a) and (b) are defined. We include 95% confidence intervals obtained via parametric bootstrap with 1000 replications, drawing the model parameters from the asymptotic multivariate normal distribution of the estimator defined by the estimated coefficients and variance‐covariance matrix and recomputing the patient‐loss measures in each replication. 95% confidence intervals are constructed using the percentiles of the resulting bootstrap distribution. *p<0.1; **p<0.05; ***p<0.01.

The nesting parameter is estimated at λ=0.245(p<0.001), inside the unit interval, confirming the consistency of the model with random utility maximisation. This also confirms that the two non‐surgical treatments are closer substitutes for one another than for surgery. A likelihood‐ratio (LR) test between the nested logit and a multinomial logit rejects the restriction λ=1 ($\chi^{2}\left(1\right)=60.11$, p<0.001).

Panel B of Table 4 reports the implied patient loss estimates. To make these patient loss estimates comparable, we focus on the subsample of participants who were randomly assigned to the full set of treatments J, for whom both patient loss measures are straightforward to compute. By contrast, for the subsample assigned to the constrained set of treatments C, it is unclear how to evaluate $\Delta {\tilde{PL}}_{i}\left(J,J\right)$ without making additional assumptions.

The loss from incorrect beliefs about post‐surgery outcomes is modest, with mean estimates of −1.12 weeks (evaluated at J) and −0.89 weeks (evaluated at C). The loss from limited availability of physiotherapy, equivalent to its exclusion from the set of available treatments, is considerably larger, with a mean of −4.13 weeks. For comparison, the NHS Constitution states that patients should wait at most 18 weeks from GP referral to treatment for elective procedures such as the one in our experiment (Pope 2023). Relative to this benchmark, the loss from incorrect beliefs (evaluated at C) is equivalent to roughly 5% of the maximum expected waiting time, whereas the loss from limited availability of physiotherapy is roughly 23%. Combining both sources, the total expected loss is estimated at −5.02 weeks (95% CI: −13.55 to −2.72), of which the limited availability of physiotherapy accounts for the large majority (around four‐fifths) and incorrect beliefs for the remainder. We stress that this comparison reflects the specific case of including or excluding physiotherapy in the set of treatments in the context of a hip replacement procedure, so the magnitude of the loss due to limited availability of treatments may differ substantially for other excluded treatments or in other clinical contexts.

Although the confidence intervals are wide, particularly for the loss from limited availability of physiotherapy, they remain bounded away from zero, indicating that the estimated patient losses are statistically significant despite the imprecision in their magnitude.

#### 5.2.2 Patient Loss by Deviations in Expected Post‐Surgery Outcomes

In Table 5, we examine some of the heterogeneity and potential mechanisms behind our patient loss estimates. We start by exploring the individual contribution of belief deviations to each of the measures of patient loss in Table 4. We estimate the following regression:

(11) $$\Delta PL_{i}=\gamma_{0}+\gamma_{1}|{\text{Stay}}_{i}^{\text{Dev}}|+\gamma_{2}|{\text{Readmission}}_{i}^{\text{Dev}}|+\gamma_{3}|{\text{Death}}_{i}^{\text{Dev}}|+u_{i},$$

where $\Delta PL_{i}$ generically denotes one of the measures of i’s patient loss, and each deviation is the absolute value of the elicited belief minus the corresponding benchmark probability for a post‐surgery outcome.

**TABLE 5:** Patient loss by deviations in expected post‐surgery outcomes.

	(1)	(2)	(3)	(4)
	$\Delta {\tilde{PL}}_{i}\left(J,J\right)$	$\Delta {\tilde{PL}}_{i}\left(C,C\right)$	$\Delta PL_{i}\left(C,J\right)$	$\Delta {\tilde{PL}}_{i}\left(C,J\right)$
	Incorrect beliefs	Incorrect beliefs	No physio	Total
$|{\text{Stay}}^{\text{Dev}}|$	−0.012***	−0.011***	−0.010***	−0.022***
	(0.004)	(0.004)	(0.003)	(0.005)
$|{\text{Readmission}}^{\text{Dev}}|$	−0.108***	−0.086***	−0.001	−0.087***
	(0.006)	(0.005)	(0.003)	(0.006)
$|{\text{Death}}^{\text{Dev}}|$	−0.175***	−0.150***	−0.004	−0.154***
	(0.020)	(0.020)	(0.003)	(0.019)
Constant	1.517***	1.313***	−3.904***	−2.592***
	(0.147)	(0.146)	(0.067)	(0.155)
$R^{2}$	0.83	0.79	0.02	0.70
Obs	928	928	928	928

*Note:* Regressors are absolute deviations between expected and benchmark post‐surgery outcomes, defined as elicited beliefs minus the corresponding benchmark values. Robust standard errors in parentheses. *p<0.1; **p<0.05; ***p<0.01.

The results in Table 5 show that elicited beliefs further away from benchmark values significantly increase the loss from incorrect beliefs in Columns (1) and (2). By contrast, they have much smaller effects on the loss from limited availability of physiotherapy in Column (3): only the coefficient on extended hospital stay is statistically significant, and it is small. All three deviations enter with the expected negative sign: a larger absolute deviation from the benchmark, for any outcome, lowers the patient‐loss measure, that is, increases the loss in absolute value. The deviations explain the bulk of the variation in the belief‐based losses, with goodness‐of‐fit measures of 0.83 (Column (1)) and 0.79 (Column (2)), whereas they have almost no explanatory power over the loss from limited availability of physiotherapy ($R^{2}=0.02$, Column (3)), which by construction does not depend on belief accuracy.

Incorrect beliefs about death have the largest effect. A one p.p. absolute deviation in the probability of death increases the total loss in Column (4) by the equivalent of about 0.15 weeks of waiting time. Deviations in beliefs about hospital readmission also have substantial effects (0.11 weeks in Column (1) and 0.09 weeks in Column (2)), while deviations in beliefs about extended hospital stay have comparatively smaller but still significant effects (0.01 weeks in Columns (1) and (2)). When we consider total patient loss in Column (4), all three deviations are statistically significant, with death the largest (0.15 weeks), followed by hospital readmission (0.09 weeks) and extended hospital stay (0.02 weeks). The $R^{2}$ of 0.70 for total patient loss indicates that these belief deviations explain around 70% of the overall variation in patient loss across participants.

#### 5.2.3 Patient Loss, Information Intervention, and Demographics

We examine the explanatory power of the information intervention and patient demographics for the loss estimates using the following regression:

$$\Delta PL_{i}=\gamma_{0}+\gamma_{1}{\text{Intervention}}_{i}+\gamma_{2}{\text{Demographics}}_{i}+u_{i}.$$

The odd‐numbered columns in Table 6 show estimation results for each patient loss measure from a specification with $\gamma_{2}=0$. The information intervention has a negative and significant coefficient on the loss from incorrect beliefs (Columns (1) and (3)) but no impact on the loss from limited availability of physiotherapy (Column (5)). Since patient losses are measured as negative values, participants who were assigned to the information intervention experienced larger losses in absolute value by approximately 0.45 weeks (Column (1)) and 0.36 weeks (Column (3)) compared to those who were not assigned to the intervention.

**TABLE 6:** Patient loss, information intervention, and education.

	(1)	(2)	(3)	(4)	(5)	(6)	(7)	(8)
	$\Delta {\tilde{PL}}_{i}\left(J,J\right)$	$\Delta {\tilde{PL}}_{i}\left(J,J\right)$	$\Delta {\tilde{PL}}_{i}\left(C,C\right)$	$\Delta {\tilde{PL}}_{i}\left(C,C\right)$	$\Delta PL_{i}\left(C,J\right)$	$\Delta PL_{i}\left(C,J\right)$	$\Delta {\tilde{PL}}_{i}\left(C,J\right)$	$\Delta {\tilde{PL}}_{i}\left(C,J\right)$
	Incor. Beli.	Incor. Beli.	Incor. Beli.	Incor. Beli.	No physio	No physio	Total	Total
Intervention	−0.445**		−0.361**		0.014		−0.346*	
	(0.203)		(0.174)		(0.068)		(0.190)	
Highly educated		0.581***		0.456***		−0.058		0.398**
		(0.205)		(0.175)		(0.068)		(0.192)
Constant	−0.906***	−1.439***	−0.717***	−1.141***	−4.140***	−4.101***	−4.856***	−5.242***
	(0.122)	(0.162)	(0.103)	(0.137)	(0.047)	(0.050)	(0.115)	(0.151)
$R^{2}$	0.01	0.01	0.01	0.01	0.00	0.00	0.00	0.01
Obs	928	928	928	928	928	928	928	928

*Note:* Columns (1)–(2) use $\Delta {\tilde{PL}}_{i}\left(J,J\right)$, the patient loss from incorrect beliefs evaluated at the full set of treatments J. Columns (3)–(4) use $\Delta {\tilde{PL}}_{i}\left(C,C\right)$, the patient loss from incorrect beliefs evaluated at the constrained set of treatments C. Columns (5)–(6) use $\Delta PL_{i}\left(C,J\right)$, the patient loss from limited availability of treatments, here the exclusion of physiotherapy from C. Columns (7)–(8) use $\Delta {\tilde{PL}}_{i}\left(C,J\right)$, the total expected patient loss. Information intervention equals 1 for participants with $I_{i}=1$ and zero otherwise. Highly educated equals 1 for participants with an undergraduate or a postgraduate degree and zero otherwise. Robust standard errors in parentheses. *p<0.1; **p<0.05; ***p<0.01.

This pattern is consistent with the results in Table 5, where larger absolute deviations from benchmark post‐surgery outcomes are associated with greater patient losses, once combined with the heterogeneous effects of the information intervention documented in Section 5.1. As shown in Figure 5, the intervention shifts the entire distribution of beliefs upward for all post‐surgery outcomes. This shift has differential effects on belief accuracy depending on participants' initial positions: for extended hospital stay, where the control group underestimates the benchmark value, the shift moves participants closer to the benchmark value on average; for hospital readmission and death, where the control group already overestimates, the shift pushes beliefs further from the benchmark on average. Because the amplified overestimation of hospital readmission and death dominates, the information intervention increases the overall patient loss due to incorrect beliefs. The effect carries through to total patient loss, which increases in absolute value by 0.35 weeks (Column (7), p<0.1). This reinforces the message of Section 5.1 that providing profile‐specific forecasts of post‐surgery outcomes does not uniformly improve belief accuracy or patient welfare.

We also investigate the explanatory power of demographics on patient loss. We do not obtain significant estimates of $\gamma_{2}$ by age group, gender, ethnic background, or employment status. Nevertheless, we obtain significant estimates for high education: highly educated participants (undergraduate or postgraduate degree) experienced smaller losses, as shown in Columns (2), (4), and (8) of Table 6. The estimates of 0.58, 0.46, and 0.40 weeks for $\gamma_{2}$ indicate that higher education is associated with belief‐based and total patient losses that are roughly half a week smaller. This finding complements the results in Section 5.1, where higher education is found to be associated with smaller belief deviations for hospital readmission and death—the two outcomes that participants overestimate on average—suggesting that more educated participants hold beliefs closer to the benchmark values for these two outcomes regardless of intervention assignment. The limited variation in some demographic characteristics (e.g., 93% of White British) may explain the lack of significant estimates for other demographics. Further research is required to investigate the impact on different critical subpopulations of interest, such as ethnic or underrepresented minorities.

## 6 Conclusions

As surgical procedures become more accessible to the general public, older and higher‐risk patients are increasingly opting for such procedures (S. Shaw et al. 2020). For these patients, the decision to undergo surgery comes with a higher risk of medical complications due to their age and comorbidities, making uncertainty about post‐surgery outcomes particularly important. In particular, one in three high‐risk patients undergoing surgery experiences serious medical complications that can lead to long‐term deterioration in their health (OSIRIS 2018; ISOS 2016; Fowler et al. 2023).

We study decision‐making for hip replacement in the UK and focus on the patient side of the decision process. We randomly allocated participants to two experimental interventions. Participants assigned to the information intervention received profile‐specific forecasts of post‐surgery outcomes. The forecasts were based on NHS Hospital Episode Statistics (HES) data for the high‐risk clinical profile used in the experiment. The information provision mimicked the idea of using predictive AI in the form of clinical decision support systems (CDSS) to assist doctors and patients in shared decision‐making. Importantly, the NHS does not typically offer such profile‐specific forecasts for elective procedures when discussing surgical treatments with patients.

In the treatment availability intervention, we experimentally modified the set of available treatments from which participants could choose by adding physiotherapy, a less invasive and non‐surgical alternative to hip replacement. This intervention allows us to study the policy implications of actively expanding treatment alternatives for patients who may not benefit from the standard surgical procedure and to measure the patient loss from limited availability of treatments.

Our results show that patient beliefs systematically differ from benchmark post‐surgery outcomes based on HES data. The information intervention has heterogeneous effects on belief accuracy: it improves alignment for extended hospital stay, where the control group underestimates the benchmark probability on average, but it amplifies overestimation for hospital readmission within 6 months and death within 90 days, where the control group already overestimates the benchmark probabilities on average.

Furthermore, welfare calculations based on a nested logit that models substitution between the non‐surgical treatments suggest that facing a constrained set of treatments that excludes physiotherapy as a non‐surgical alternative generates larger average patient losses than incorrect beliefs about post‐surgery outcomes, though the magnitude of the losses due to limited availability of treatments may differ across other excluded treatments or in other clinical contexts. We also find evidence that highly educated participants tend to incur smaller losses from incorrect beliefs.

Current principles of informed consent for surgery in the UK emphasise a legal obligation to inform patients about all risks and alternatives that are “material” to their decision, with limited scope for clinician judgement in selecting relevant information. However, little information or guidance exists to determine which risks and benefits are material or meaningful to individual patients, or how to support their decision‐making while meeting legal requirements and ensuring healthcare resources are directed to those who might most benefit. We know very little about quantifying possible outcomes of surgery and other treatments in ways that provide actionable information and assist patients' decision‐making. This research highlights this knowledge gap, emphasising that information may need to be aligned with patient priorities. Further research integrating methodologies across the medical, economic, and social sciences is required to understand approaches that meet both legal requirements and patient needs, enabling appropriate and cost‐effective treatment.

Moreover, our results have implications for the design and deployment of CDSS that use AI‐enabled predictions to improve shared decision‐making. While such tools hold promise for reducing uncertainty about post‐surgery outcomes, our findings suggest that simply providing profile‐specific forecasts does not uniformly improve belief accuracy. This underscores the need to carefully evaluate how information about post‐surgery outcomes is framed and communicated. Our findings also highlight the importance of offering non‐surgical alternatives to high‐risk patients who may not benefit from standard surgical procedures. The substantial welfare losses associated with limited availability of treatments suggest that expanding the range of treatments presented during consultations, such as including physiotherapy alongside surgery, could yield meaningful improvements in patient welfare, particularly for older patients with comorbidities for whom surgical risks are elevated.

Several limitations of our study should be noted. First, our binary information intervention does not allow us to fully isolate the effect of providing forecasts for a specific high‐risk clinical profile from risk salience. Because the control arm provides general NHS risk framing while the intervention provides quantified, profile‐specific forecasts of post‐surgery outcomes, the observed treatment effect may reflect both the provision of specific numerical information and the use of forecasts for Sam's clinical profile. A three‐arm design, adding an arm with quantified average‐patient statistics, would enable this decomposition and represents an important direction for future work. Second, the experiment uses a vignette‐based design in which participants imagine themselves as a hypothetical patient, which may not fully capture the emotional and cognitive processes involved in real clinical decisions. Third, our experiment focuses exclusively on a hip replacement procedure, and our sample is predominantly female and white British, which may limit generalisability to the broader NHS surgical population and treatments. Despite these limitations, the experimental design contributes to the debate on a real‐world policy question: whether CDSS that provide profile‐specific forecasts of post‐surgery outcomes can improve patient decision‐making in the NHS.

## Author Contributions

**Francesca Cornaglia:** conceptualization, formal analysis, funding acquisition, writing – review and editing. **Alessandro Iaria:** conceptualization, formal analysis, funding acquisition, methodology, writing – original draft, writing – review and editing. **Eugenio Felipe Merlano:** conceptualization, formal analysis, methodology, writing – original draft, writing – review and editing. **John R. Prowle:** conceptualization, formal analysis, funding acquisition, writing – review and editing.

## Funding

We acknowledge financial support from the National Institute for Health and Care Research (NIHR) through the OSIRIS (Optimizing Shared decision‐makIng for high‐ RIsk major Surgery) program (Award ID: RP‐PG‐0218‐20001).

## Conflicts of Interest

The authors declare no conflicts of interest.

## Data Availability

The data that support the findings of this study are available on request from the corresponding author. The data are not publicly available due to privacy or ethical restrictions.

## Endnotes

^1^
Although standard in individual‐level discrete choice models (given the complexity of implementing instrumental variables in the context of maximum likelihood), exogeneity of the set of treatments and of the regressors could in theory be partially relaxed by means of a control function approach (Aguirregabiria et al. 2023; Petrin and Train 2010). Given the additional complexity of potentially incorrect beliefs we consider, in our setting it is however unclear whether one could specify an appropriate control function. As we discuss in detail below, we instead address these endogeneity issues by imposing various dimensions of randomisation in our online experiment.

^2^
This nesting choice is motivated by the evidence from our online experiment, as we show in Figure 6 below.

^3^
In extensive robustness checks, we investigated mixed logit versions of the model. However, the credible estimation of a mixed logit model typically requires repeated treatment choices per respondent (e.g., Revelt and Train 1998), while our data are cross‐sectional. Due to this data limitation, we found our estimates of mixed logit versions of the model not robust and opted for a more parsimonious nested logit model.

^4^
The information intervention combined two elements: it made average‐patient probabilities explicit and showed profile‐specific deviations from these averages for the clinical profile described in the medical scenario. Our estimates therefore capture the combined effect of these elements rather than separately identifying the effect of quantification from the effect of profile‐specific deviations.

^5^
This sampling scheme was developed in collaboration with clinical researchers experienced in perioperative medicine and critical care.

^6^
The main charities and institutions include: Barts Health NHS Trust, Queen Mary University of London, Versus Arthritis, Bowel Research UK, and Patient Safety Learning.

^7^
We collected a total of 2074 observations.

^8^
In Appendix Table A3, we report the corresponding parameter and patient loss estimates for a multinomial logit version of the model (a model that constrains λ=1).

## Appendix A Derivation of Patient Surplus and Loss Measures

In this Appendix, we derive the patient surplus and loss measures (5)–(9). We use the notation introduced in Section 2, including $D_{i}\left(x,A\right)$ and $d_{iS}$. We also use the same convention that tilded surplus and loss objects involve treatment choices made using expected post‐surgery outcomes, while untilded objects are evaluated only at benchmark post‐surgery outcomes. For further discussions on the model and its associated welfare implications, see Train (2015). In each derivation, the expected‐maximum (log‐sum) terms $E_{\varepsilon }\left[\max_{k\in A}\left\{U_{ik}\right\}\right]=\ln D_{i}\left(c_{S},A\right)$ and $E_{\varepsilon }\left[\max_{k\in A}\left\{EU_{ik}\right\}\right]=\ln D_{i}\left({\tilde{c}}_{iS},A\right)$ are expressed net of the Euler constant, as in Section 2. Equation (5) can be obtained as:

$$\begin{array}{c} {\tilde{PS}}_{ij^{⋆}}\left(J\right)=-\frac{1}{\alpha }E_{\varepsilon }\left[U_{ij^{⋆}}\right]\\ =-\frac{1}{\alpha }E_{\varepsilon }\left[EU_{ij^{⋆}}+d_{ij^{⋆}}\right]\\ =-\frac{1}{\alpha }\left(E_{\varepsilon }\left[\max_{k\in J}\left\{EU_{ik}\right\}\right]+d_{iS}\mathrm{Pr}\left[S|J,{\tilde{c}}_{iS}\right]\right)\\ =-\frac{1}{\alpha }\left(\ln\;D_{i}\left({\tilde{c}}_{iS},J\right)+d_{iS}\mathrm{Pr}\left[S|J,{\tilde{c}}_{iS}\right]\right). \end{array}$$

Equation (6) is obtained as:

$$\begin{array}{c} \Delta {\tilde{PL}}_{i}\left(J,J\right)={\tilde{PS}}_{ij^{⋆}}\left(J\right)-PS_{ir^{⋆}}\left(J\right)\\ =-\frac{1}{\alpha }\left(E_{\varepsilon }\left[EU_{ij^{⋆}}+d_{ij^{⋆}}\right]-E_{\varepsilon }\left[U_{ir^{⋆}}\right]\right)\\ =-\frac{1}{\alpha }\left(E_{\varepsilon }\left[\max_{k\in J}\left\{EU_{ik}\right\}\right]+d_{iS}\mathrm{Pr}\left[S|J,{\tilde{c}}_{iS}\right]-E_{\varepsilon }\left[\max_{k\in J}\left\{U_{ik}\right\}\right]\right)\\ =-\frac{1}{\alpha }\left(\ln\;D_{i}\left({\tilde{c}}_{iS},J\right)-\ln\;D_{i}\left(c_{S},J\right)+d_{iS}\mathrm{Pr}\left[S|J,{\tilde{c}}_{iS}\right]\right). \end{array}$$

Equation (7) is derived as:

$$\begin{array}{c} PS_{ir^{c}}\left(C\right)=-\frac{1}{\alpha }E_{\varepsilon }\left[U_{ir^{c}}\right]\\ =-\frac{1}{\alpha }E_{\varepsilon }\left[\max_{k\in C}\left\{U_{ik}\right\}\right]\\ =-\frac{1}{\alpha }\ln\;D_{i}\left(c_{S},C\right). \end{array}$$

From this, it follows that Equation (8) can be derived as:

$$\begin{array}{c} \Delta PL_{i}\left(C,J\right)=PS_{ir^{c}}\left(C\right)-PS_{ir^{⋆}}\left(J\right)\\ =-\frac{1}{\alpha }\left(E_{\varepsilon }\left[U_{ir^{c}}\right]-E_{\varepsilon }\left[U_{ir^{⋆}}\right]\right)\\ =-\frac{1}{\alpha }\left(E_{\varepsilon }\left[\max_{k\in C}\left\{U_{ik}\right\}\right]-E_{\varepsilon }\left[\max_{k\in J}\left\{U_{ik}\right\}\right]\right)\\ =-\frac{1}{\alpha }\left(\ln\;D_{i}\left(c_{S},C\right)-\ln\;D_{i}\left(c_{S},J\right)\right). \end{array}$$

Finally, using the analogous definition of ${\tilde{PS}}_{ij^{c}}\left(C\right)$ with J replaced by C, Equation (9) can be obtained as:

$$\begin{array}{c} \Delta {\tilde{PL}}_{i}\left(C,J\right)={\tilde{PS}}_{ij^{c}}\left(C\right)-PS_{ir^{c}}\left(C\right)+PS_{ir^{c}}\left(C\right)-PS_{ir^{⋆}}\left(J\right)\\ ={\tilde{PS}}_{ij^{c}}\left(C\right)-PS_{ir^{⋆}}\left(J\right)\\ =-\frac{1}{\alpha }\left(E_{\varepsilon }\left[EU_{ij^{c}}+d_{ij^{c}}\right]-E_{\varepsilon }\left[U_{ir^{⋆}}\right]\right)\\ =-\frac{1}{\alpha }\left(E_{\varepsilon }\left[\max_{k\in C}\left\{EU_{ik}\right\}\right]+d_{iS}\mathrm{Pr}\left[S|C,{\tilde{c}}_{iS}\right]-E_{\varepsilon }\left[\max_{k\in J}\left\{U_{ik}\right\}\right]\right)\\ =-\frac{1}{\alpha }\left(\ln\;D_{i}\left({\tilde{c}}_{iS},C\right)-\ln\;D_{i}\left(c_{S},J\right)+d_{iS}\mathrm{Pr}\left[S|C,{\tilde{c}}_{iS}\right]\right). \end{array}$$

## Appendix B Online Experiment and Additional Results

**TABLE A1:** Demographic characteristics by intervention group (Obs = 1877).

	$I_{i}=0$, C	$I_{i}=0$, J	$I_{i}=1$, C	$I_{i}=1$, J	*p*‐value
	Obs = 475	Obs = 481	Obs = 474	Obs = 447	
Age group					0.907
65−	153 (32.2%)	164 (34.1%)	162 (34.2%)	148 (33.1%)	
65+	322 (67.8%)	317 (65.9%)	312 (65.8%)	299 (66.9%)	
Female					0.754
0	74 (15.6%)	67 (13.9%)	70 (14.8%)	59 (13.2%)	
1	401 (84.4%)	414 (86.1%)	404 (85.2%)	388 (86.8%)	
White British					0.681
0	37 (7.79%)	31 (6.44%)	29 (6.12%)	34 (7.61%)	
1	438 (92.2%)	450 (93.6%)	445 (93.9%)	413 (92.4%)	
Education level					0.741
Low	203 (42.7%)	216 (44.9%)	214 (45.1%)	202 (45.2%)	
Medium	102 (21.5%)	116 (24.1%)	100 (21.1%)	94 (21.0%)	
High	170 (35.8%)	149 (31.0%)	160 (33.8%)	151 (33.8%)	
Employment					0.967
Working	102 (21.5%)	105 (21.8%)	100 (21.1%)	91 (20.4%)	
Retired	336 (70.7%)	335 (69.6%)	333 (70.3%)	324 (72.5%)	
Other	37 (7.79%)	41 (8.52%)	41 (8.65%)	32 (7.16%)	
Arthritis	0.39	0.41	0.43	0.41	0.683

*Note:* Columns represent intervention groups by information intervention (baseline information, $I_{i}=0$; profile‐specific forecasts, $I_{i}=1$) and treatment availability intervention (constrained set C or full set J). The constrained set C excludes physiotherapy. The Arthritis row reports the group mean, that is, the share of participants reporting arthritis. Education levels are defined as low (less than an undergraduate degree), medium (undergraduate degree), and high (postgraduate degree).

**TABLE A2:** Beliefs and information intervention—robustness.

	${Stay}^{\text{Dev}}$		${Readmission}^{\text{Dev}}$		${Death}^{\text{Dev}}$	
	(1)	(2)	(3)	(4)	(5)	(6)
Intervention	$6.190^{***}$	$6.178^{***}$	$8.226^{***}$	$8.159^{***}$	$1.311^{***}$	$1.290^{**}$
	(0.928)	(0.929)	(0.763)	(0.762)	(0.506)	(0.503)
Age 60‐69		0.792		1.804		0.361
		(1.535)		(1.235)		(0.726)
Age 70‐79		2.003		1.985		$1.507^{*}$
		(1.696)		(1.368)		(0.840)
Age 80+		$5.016^{*}$		$4.514^{*}$		1.960
		(2.745)		(2.336)		(1.625)
Female		$2.884^{**}$		1.379		−0.040
		(1.352)		(1.071)		(0.869)
White British		−0.307		−0.949		−0.543
		(2.006)		(1.700)		(1.212)
Retired		−0.238		0.151		−0.341
		(1.351)		(1.125)		(0.690)
Employment:other		0.293		2.776		0.052
		(1.933)		(1.687)		(0.943)
Medium edu		−$2.774^{**}$		−$3.806^{***}$		−$2.811^{***}$
		(1.229)		(0.972)		(0.633)
High edu		−$3.172^{***}$		−$3.293^{***}$		−$2.490^{***}$
		(1.070)		(0.887)		(0.578)
Arthritis		−1.034		−0.264		0.351
		(0.974)		(0.806)		(0.543)
Constant	−$10.006^{***}$	−$11.287^{***}$	$4.183^{***}$	$3.909^{*}$	$3.982^{***}$	$5.254^{***}$
	(0.675)	(2.697)	(0.508)	(2.057)	(0.370)	(1.626)
$R^{2}$	0.023	0.034	0.059	0.075	0.004	0.023
Obs	1877	1877	1877	1877	1877	1877

*Note:* Dependent variables are deviations between expected and benchmark post‐surgery outcomes, defined as elicited beliefs minus the corresponding benchmark probabilities. Both elicited and benchmark probabilities are measured on a 0‐100 scale. Odd columns replicate Table 3; even columns include demographic controls. Reference categories: Age 50–59, Male, Non‐White British, Employed, Low education. Education levels are defined as low (less than an undergraduate degree), medium (undergraduate degree), and high (postgraduate degree). Robust standard errors in parentheses. *p<0.1; **p<0.05; ***p<0.01.

**TABLE A3:** Multinomial logit and patient loss estimates.

Panel A: Model estimates (Obs = 1877)						
				Expected post‐surgery outcomes $\left({\tilde{c}}_{iS}\right)$		
	GP $\left(\delta_{G}\right)$	Physio $\left(\delta_{P}\right)$	Waiting (α)	Stay	Readmission	Death
Estimate	−2.907***	−1.814***	−0.016*	0.002	−0.017***	−0.017***
SE	(0.155)	(0.122)	(0.008)	(0.004)	(0.005)	(0.006)

**Panel B: Patient loss estimates**						
	**Obs**	**Mean**	**Std. Dev.**	**[95% CI low**	**95% CI upper]**	
(a) Expected loss from incorrect beliefs about post‐surgery outcomes						
$\Delta {\tilde{PL}}_{i}\left(J,J\right)$	928	−1.47	3.87	−8.10	−0.29	
$\Delta {\tilde{PL}}_{i}\left(C,C\right)$	928	−0.81	2.60	−4.70	−0.15	
(b) Expected loss from limited availability of treatments						
$\Delta PL_{i}\left(C,J\right)$	928	−10.51	1.44	−53.04	−3.43	
Total expected loss: (a) + (b)						
$\Delta {\tilde{PL}}_{i}\left(C,J\right)$	928	−11.33	3.03	−56.59	−3.83	

*Note:* Panel A presents the estimated coefficients for the multinomial logit model. Panel B presents: (a) the patient loss from incorrect beliefs about post‐surgery outcomes, (b) the patient loss from limited availability of treatments (the exclusion of physiotherapy from the constrained set C), and the total expected patient loss, all evaluated on the subsample assigned to the full set of treatments J (Obs = 928) for which both (a) and (b) are defined. We include 95% confidence intervals obtained via parametric bootstrap with 1000 replications, drawing the model parameters from the asymptotic multivariate normal distribution of the estimator defined by the estimated coefficients and variance‐covariance matrix and recomputing the patient‐loss measures in each replication. Bootstrap replications yielding near‐zero waiting coefficients (|α|<0.001) are discarded to ensure numerical stability in the patient loss calculations. 95% confidence intervals are constructed using the percentiles of the resulting bootstrap distribution. *p<0.1; **p<0.05; ***p<0.01.
